# Maternal α-casein deficiency extends the lifespan of offspring and programmes their body composition

**DOI:** 10.1007/s11357-024-01273-2

**Published:** 2024-07-12

**Authors:** Andreas F. Kolb, Claus Mayer, Alina Zitskaja, Linda Petrie, Khulod Hasaballah, Claire Warren, Ailsa Carlisle, Simon Lillico, Bruce Whitelaw

**Affiliations:** 1 Nutrition, Obesity and Disease Research Theme, Rowett Institute, University of Aberdeen, Aberdeen, AB25 2ZD Scotland; 2 Biomathematics and Statistics Scotland (BioSS), University of Aberdeen, Aberdeen, AB25 2ZD Scotland; 3 Roslin Institute, University of Edinburgh, Edinburgh, Scotland

**Keywords:** Perinatal nutrition, Growth, Programming, Caseins, Lipid Metabolism, Longevity, Body Composition, Adipose Tissue, Body Size, Gene Expression Profiling, Body Weight

## Abstract

Early nutrition has significant effects on physiological outcomes during adult life. We have analysed the effect of maternal α-casein (CSN1S1) deficiency on the physiological fate of dams and their offspring. α-casein deficiency reduces maternal milk protein concentration by more than 50% and attenuates the growth of pups to 27% (*p* < 0.001) of controls at the point of weaning. This is associated with a permanent reduction in adult body weight (− 31% at 25 weeks). Offspring nursed by α-casein deficient dams showed a significantly increased lifespan (+ 20%, χ^2^: 10.6; *p* = 0.001). Liver transcriptome analysis of offspring nursed by α-casein deficient dams at weaning revealed gene expression patterns similar to those found in dwarf mice (reduced expression of somatotropic axis signalling genes, increased expression of xenobiotic metabolism genes). In adult mice, the expression of somatotropic axis genes returned to control levels. This demonstrates that, in contrast to dwarf mice, attenuation of the GH-IGF signalling axis in offspring nursed by α-casein deficient dams is transient, while the changes in body size and lifespan are permanent. Offspring nursed by α-casein deficient dams showed permanent changes in body composition. Absolute and relative adipose tissue weights (*p* < 0.05), the percentage of body fat (*p* < 0.001) as well as adipocyte size in epididymal white adipose tissue are all reduced. Serum leptin levels were 25% of those found in control mice (*p* < 0.001). Liver lipid content and lipid composition were significantly altered in response to postnatal nutrition. This demonstrates the nutrition in early life programmes adult lipid metabolism, body composition and lifespan.

**Supplementary Information:**

The online version contains supplementary material available at 10.1007/s11357-024-01273-2.

## Introduction

Many studies in mammalian model systems have demonstrated that early nutrition is a major determinant of adult health outcomes [1, 58]. Many of these experiments assessed the consequences of intrauterine growth deficiency (often elicited by a protein-deficient diet fed to the mother) or by intrauterine overfeeding (by exposing the mother to a high-calorie diet) [56]. Both interventions sensitise the offspring to later metabolic challenges like a high-fat or high-sugar diet. Offsprings exposed to intrauterine under- or overfeeding are typically more prone to metabolic health problems, including obesity and type 2 diabetes (or insulin insensitivity) [31, 55].

Nutrition in the immediate postnatal period, i.e. during lactation, has also been shown to be important for adult health outcomes. Typically, overfeeding during early postnatal life is associated with worse metabolic health outcomes, while attenuated growth during the early postnatal period is associated with metabolic health improvements [56]. The Barker hypothesis states that small birth weight is associated with higher susceptibility to metabolic disease [33]. The assumption is that this is mediated by catch-up growth during early postnatal life. The data derived from cohorts of the Dutch Hunger Winter illustrate the importance of postnatal nutrition [38, 66].

This effect of postnatal nutrition has been studied extensively in human cohorts, and the typical comparison analysed was between bottle feeding and breastfeeding. However, in humans, many parameters affect pre- and postnatal growth, and confounding factors make it difficult to establish clear-cut mechanistic physiological links [58]. The key ingredient determining pre- and postnatal growth is protein. Human breast milk contains around 10 g/l protein with a whey-to-casein ratio of 30% to 70%. In contrast, cow’s milk contains milk protein at a concentration of around 30 g/l with a whey-to-casein ratio of 20% to 80% [65]. Milk formula tries to replicate the whey-to-casein ratio of human milk and also adjusts the protein concentration to that found in human milk [60]. An increase in the proportion of whey protein in total dietary protein has been shown to reduce body fat in animal experimentation [54].

The precise length of the postnatal period is not easy to define in humans, but in rodent model systems, it is typically equivalent to the lactation period (around 21 days in mice and rats). Attenuated growth in rodents is often achieved by manipulating litter size. Reduced litter size enables each pup to access more milk and, therefore, pups show a higher growth rate. In contrast, increased litter size reduces pup growth during lactation. An alternative intervention, which leads to attenuated pup growth during lactation, is the reduction of protein supply to the mother during lactation. This is typically achieved by reducing dietary protein from 20 to 8% [56]. Both interventions, which attenuate growth during lactation, lead to an extension of lifespan in rodents [24]. This indicates the importance of postnatal growth for lifelong health outcomes.

We have developed an alternative model to modulate pup growth during lactation by inactivating the α-casein gene in mice [44, 46]. In contrast to β-casein [48], α-casein has a functional role in the formation of the casein micelle. Therefore, α-casein deficiency not only eliminates α-casein from milk but also reduces the secretion of other casein proteins. Overall, this leads to a significant reduction in milk protein content. As a consequence, the growth of pups nursed by α-casein deficient dams during lactation is restricted to less than 40% of control pups (nursed by wild-type dams). This results in a permanent reduction of adult body weight in pups nursed by α-casein deficient dams [44, 46]. We show here that this reduced body size is associated with a significantly increased lifespan of these pups (+ 20%, χ^2^: 10.6; *p* = 0.001). The analysis of the liver transcriptome of the growth-restricted pups shows strong similarity to that of dwarf mice at day 21, but this similarity is much diminished by day 100. In addition, maternal α-casein deficiency also programmes body composition of offspring towards a reduced size of adipose tissue depots and reduced lipid storage in the liver.

## Materials and methods

### Animal experimentation

Transgenic mice were generated at Genoway-Charles River (Lyon, France) on an SV129xC57BL/6 mixed background. After transfer to the transgenic mouse facility at the Roslin Institute, the mice were maintained in accordance with Home Office guidelines. This study was approved by the Roslin Institute Animal Ethics Committee and was performed under Home Office License 60/3779. Mice were maintained on a C57B/6 background. Mice were housed in opaque stock cages with a dimension of 48 × 28 × 13 cm with bedding, environmental enrichment and nesting material. Cages were changed once per week. The environment was kept at 21 + / − 2 °C and 55 + / 15% relative air humidity with a 12-h light, 12-h dark cycle (lights off at 7 pm). Food and tap water were available ad libitum, and water bottles were routinely changed twice per week [46].

**Fig. 1:**
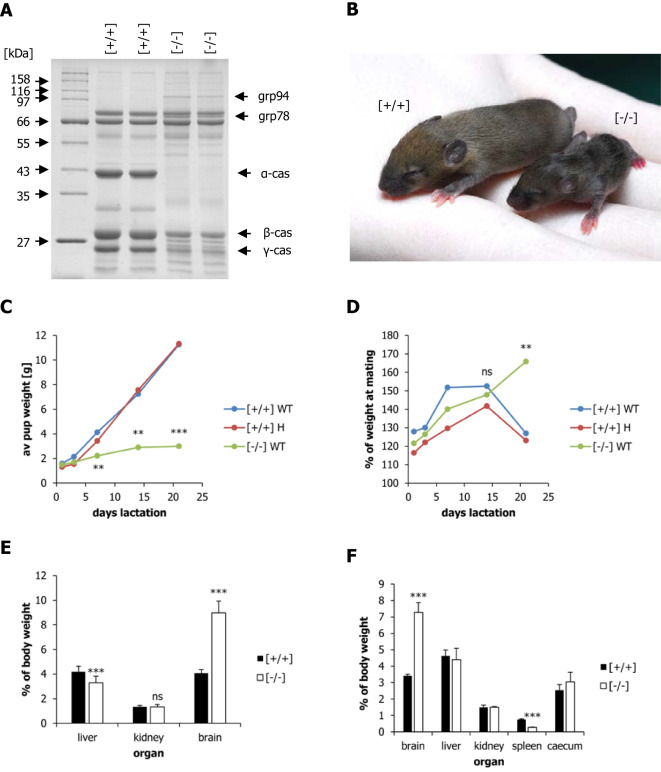
Characterisation of α-casein deficient dams and their offspring during lactation. (**A**) SDS–polyacrylamide gel analysis of milk derived from representative wild-type [+ / +] and α-casein targeted [− / −] mice. Defatted whole milk samples were separated on a 10% gel and stained with Coomassie Blue. The sizes of the protein molecular weight markers (New England Biolabs, broad range protein marker) are indicated, as are the positions of the α-casein, β-casein, γ-casein, grp78 and grp94 proteins. Grp78 and grp94 are activated by endoplasmic reticulum stress in the mammary gland of α-casein deficient mice. (**B**) Photograph of offspring nursed by wild-type [+ / +] or α-casein deficient [− / −] dams at day 15 of lactation. (**C**) Weight gain of pups (wild-type pups: WT; α-casein heterozygous pups: H) nursed by α-casein deficient [− / −] and wild-type [+ / +] dams (*n* = 3) during the lactation period. Litters were switched after birth. Note that weight gain is determined by the maternal and not by pup genotype. (**D**) Weight gain of α-casein deficient [− /−] and wild-type [+ / +] dams feeding wild-type pups (WT) or α-casein heterozygous pups (H) during the lactation period. (**E**) Percentage of liver, kidney and brain weights relative to total body weight in pups nursed by wild-type [+ / +] and α-casein deficient dams [− / −] dams at day 15 of lactation. (**F**) Percentage of brain, liver, kidney, spleen and caecum weights relative to total body weight in pups nursed by wild-type [+ / +] and α-casein deficient dams [− / −] dams at day 21 of lactation. Significance values were determined by one-way ANOVA, followed by a Bonferroni post-hoc test in Graph-Pad Prism: **p < 0.01, ***p < 0.001

**Fig. 2:**
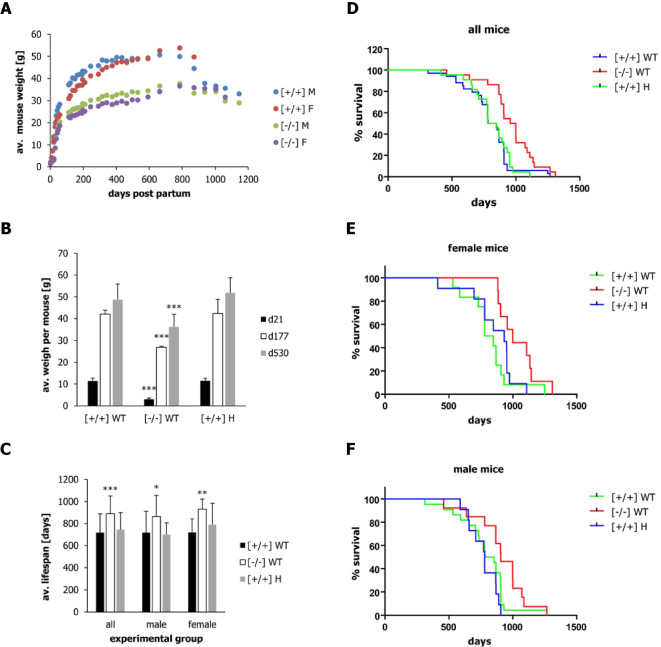
Weight development and lifespan of offspring nursed by wild-type [+ / +] and α-casein deficient [− / −] dams. (**A**) Average weight of offspring nursed by wild-type [+ / +] and α-casein deficient [− / −] dams over the entire lifespan. F indicates female mice; M indicates male mice. (**B**) Average weight of offspring (wild-type [WT] or heterozygous [H]) nursed by wild-type [+ / +] and α-casein deficient [− / −] dams at day 21 (weaning), day 177 and day 530 of life. Error bars indicate standard deviation. Significance levels are shown for a one-way ANOVA; ***p < 0.001. (**C**) Average lifespan of offspring (wild-type [WT] or heterozygous [H]) nursed by wild-type [+ / +] and α-casein deficient [− / −] dams for all, male and female mice. Significance values were determined by one-way ANOVA, followed by a Bonferroni post-hoc test in Graph-Pad Prism (see Supplementary Table ST3): *p < 0.05, **p < 0.01, ***p < 0.001. (**D–F**) Kaplan Meyer survival plots for the lifespan of offspring nursed by wild-type [+ / +] and α-casein deficient [− / −] dams. Data are shown for all (**D**), female (**E**) and male (**F**) mice. The plots were generated in Graph-Pad Prism and analysed using the log-rank Mantel–Cox test. The numerical results for the chi-squared test and the corresponding p-values are shown in Table 1

For the lifespan experiments (Figs. 1 and 2), C57B/6 wild-type and α-casein deficient dams were mated with C57B/6 wild-type males. Upon delivery, litters were swapped between the wild-type and α-casein deficient dams, such that the wild-type dams nursed heterozygous offspring and the α-casein deficient dams nursed wild-type offspring. In parallel, a control group was set up in which wild-type dams nursed their own offspring. This control group was designed to assess whether the process of cross-fostering has any impact on long-term health and survival outcomes. Three litters were used for each of the three treatments (*n* = 3) (Supplementary Table ST2 and Fig. S1). The animals were kept on a chow diet throughout the experiment.

**Fig. 3:**
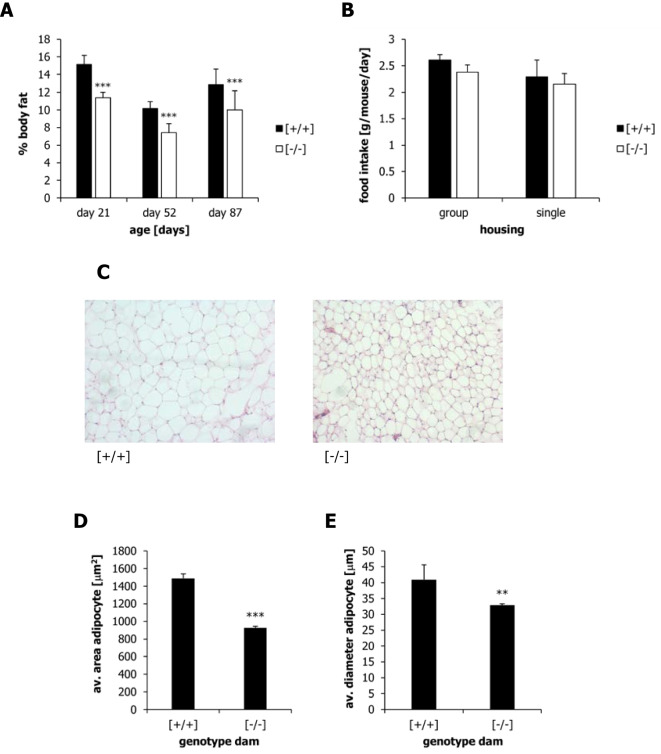
Body composition, food intake and adipocyte size of offspring nursed by wild-type [+ / +] or α-casein deficient [− / −] dams. (**A**) Percentage of body fat at day 21, day 53 and day 80 of postnatal life as measured by ECHO MRI scanning. (**B**) Food intake of offspring nursed by wild-type [+ / +] or α-casein deficient [− / −] dams during the group housing phase and the individual/single housing phase. Mice were offered ad libitum access to a synthetic control diet. (**C**) Histological staining of epididymal white adipose tissue (eWAT) using eosin and haematoxylin. Representative tissue sections of eWAT derived from offspring nursed by wild-type [+ / +] or α-casein deficient [− / −] dams. (**D**) The average area per adipocyte calculated from three independent tissue areas from three different animals (*n* = 3). (**E**) The average diameter per adipocyte calculated from three independent tissue areas from three different animals (*n* = 3). Sections were analysed by the Adiposoft plug-in for Image-J. Data were analysed using one-way ANOVA, followed by a Bonferroni post-hoc test in Graph-Pad Prism. **p < 0.01, ***p < 0.001

**Fig. 4:**
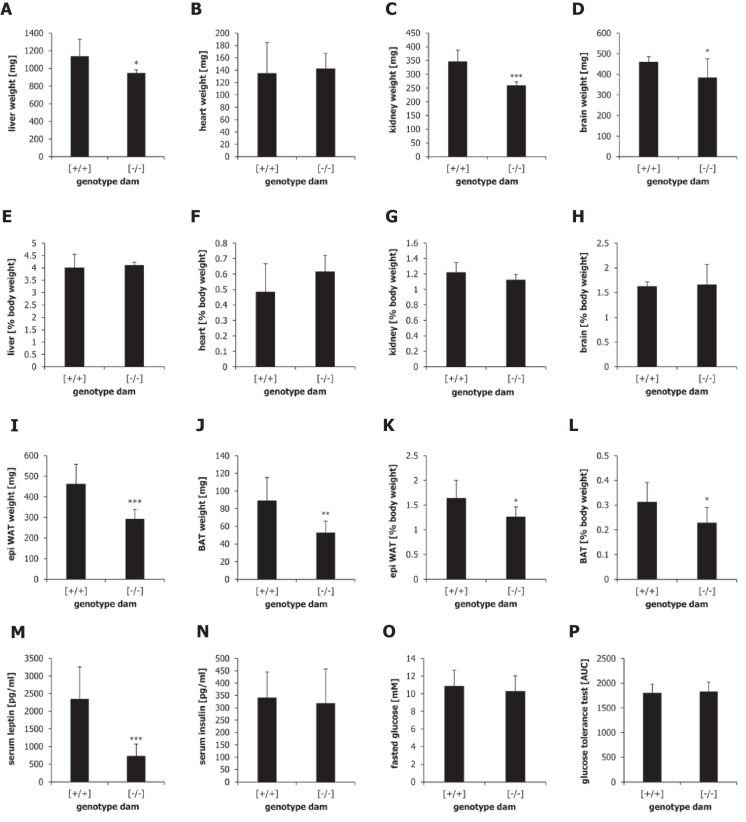
Organ weights of offspring nursed by wild-type dams [+ / +] or α-casein deficient dams [− / −] at day 100 of life. (**A**–**H**) The average weights of liver (**A**), heart (**B**), kidney (**C**) and brain (**D**) are shown in mg. The average of the organ weights relative to total body weights are shown as percentages for liver (**E**), heart (**F**), kidney (**G**) and brain (**H**). (**I**–**L**) Adipose tissue weights and serum parameters of offspring nursed by wild-type dams [+ / +] or α-casein deficient dams [− / −] at day 100 of life. The average weights of epididymal white adipose tissue (**I**) and brown adipose tissue depots (**J**) are shown in mg. The average of the adipose tissue weights relative to total body weights are shown as percentages for epididymal white adipose tissue (**K**) and brown adipose tissue (**L**). (**M**) Serum leptin concentrations in pg/ml as measured by Luminex analysis. (**N**) Serum insulin concentrations in pg/ml as measured by Luminex analysis. (**O**) Fasted glucose (in mM) measured in mice fasted for 5 h. (**P**) Area under curve derived from an intraperitoneal glucose tolerance test of fasted mice (5 h). Serum glucose levels were measured 15 min, 30 min, 60 min and 120 min post IP injection of a glucose solution (1.5 mg/g body weight). The area under the curve was quantified by an Excel add-in (Joel Usansky, University of Irvine). Data were analysed using a one-way ANOVA. *p < 0.05, **p < 0.01, ***p < 0.001

**Fig. 5:**
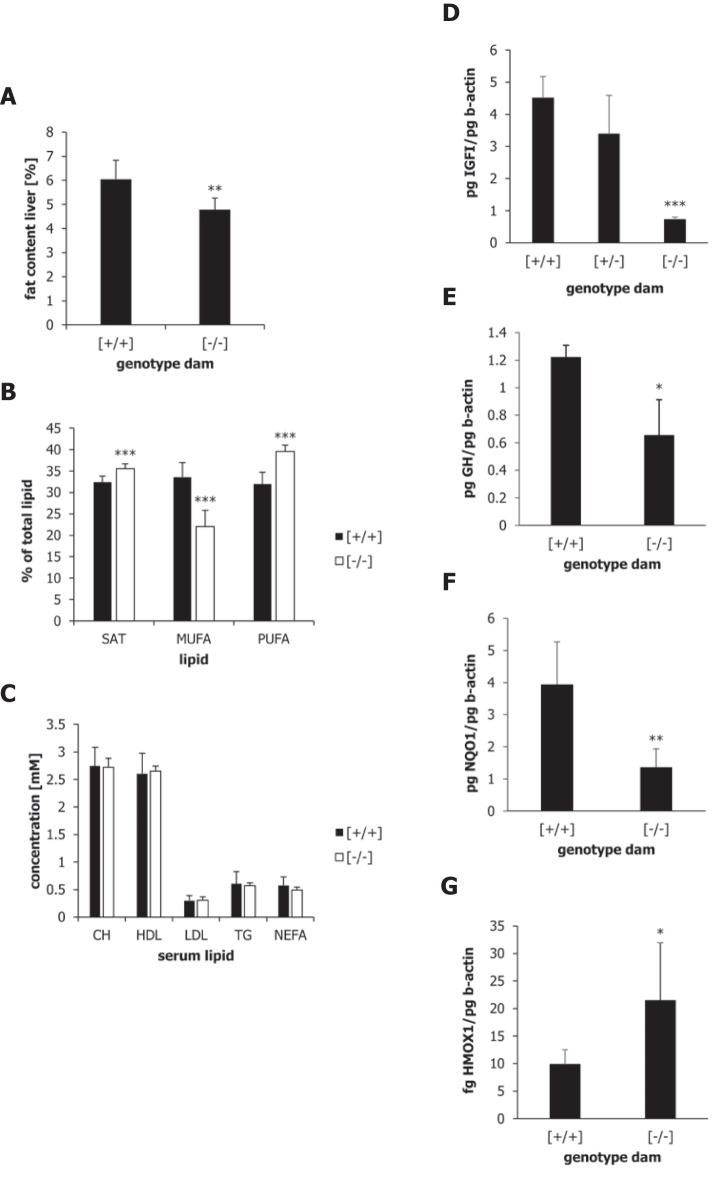
(**A**–**C**) Liver and serum lipids in offspring nursed by α-casein deficient dams [− / −] or wild-type dams [+ / +] measured at day 100. (A) Total liver lipid was analysed as described in the ‘Materials and methods’ section. Percent fat weight per liver weight is shown. (**B**) Fatty acid content in liver samples was determined as described in the methods section. Percentages of total saturated (SAT), mono-unsaturated (MUFA) and poly-unsaturated (PUFA) fatty acids are shown. (**C**) Serum lipids were determined as described in the methods section. The concentrations of free cholesterol (CH), HDL cholesterol (HDL), LDL cholesterol (LDL), total triglycerides (TG) and non-esterified fatty acids (NEFA) are shown in mM. Data were analysed by one-way ANOVA with Bonferroni post-hoc correction. **p < 0.01, ***p < 0.001. (**D**–**G**) Gene expression in the liver and pituitary gland of mice at weaning. (**D**) IGFI expression at day 21 post-partum in liver tissue of offspring (*n* = 5) nursed by wild-type [+ / +], heterozygous [+ / −] and α-casein deficient [− / −] dams. (**E**) Growth hormone (GH) expression at day 21 post-partum in pituitary tissue of offspring (*n* = 5) nursed by wild-type [+ / +] and α-casein deficient [− / −] dams. (**F**) NQO1 expression at day 21 post-partum in liver tissue of offspring (*n* = 5) nursed by wild-type [+ / +] and α-casein deficient [− / −] dams. (**G**) HMOX1 expression at day 21 post-partum in liver tissue of offspring (*n* = 5) nursed by wild-type [+ / +] and α-casein deficient [− / −] dams. Expression of all genes is shown as pg or fg per pg of reference gene β-actin. Data were analysed using one-way ANOVA. *p < 0.05, **p < 0.01, ***p < 0.001

**Fig. 6:**
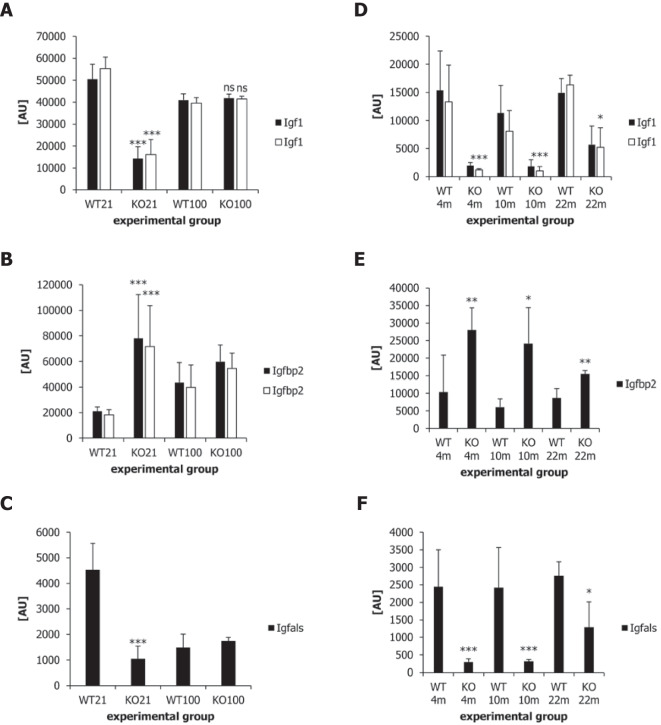
Comparison of genes of the growth hormone-IGF axis in offspring nursed by α-casein deficient dams and in Ames dwarf mice. (**A**) Expression of two probes for Igf1 at day 21 and day 100 in liver tissue of offspring nursed by α-casein deficient dams (KO) and control dams (WT). (**B**) Expression of two Igfbp2 probes at day 21 and day 100 in liver tissue of KO and WT mice. Igfbp2 acts as an inhibitor of IGF signalling. (**C**) Expression of insulin-like growth factor binding protein acid labile subunit (Igfals) at day 21 and day 100 in liver tissue of KO and WT mice. Igfals acts as an activator of IGF signalling. (**D**) Expression of Igf1 in Ames dwarf mice (KO) and control (WT) mice at the ages of 4 months, 10 months and 22 months (4m, 10m, 22m). (**E**) Expression of Igfbp2 in Ames dwarf mice (KO) and control (WT) mice at the ages of 4 months, 10 months and 22 months. (**F**) Expression of Igfals in Ames dwarf mice (KO) and control (WT) mice at the ages of 4 months, 10 months and 22 months. The comparisons between animals nursed by α-casein deficient mothers (KO) or control mothers (WT) were analysed by ANOVA. *p < 0.05, **p < 0.01, ***p < 0.001. The comparisons of gene expression between Ames and control mice were done using a two-tailed *t*-test. *p < 0.05, **p < 0.01, ***p < 0.001

For the physiological analysis (Figs. 3, 4, 5 and 6), the α-casein mutation was bred onto a CD1 background for 10 generations and transferred to the University of Aberdeen. These mice also carry an additional transgene encoding the light chain of an antiviral antibody, which does not affect the physiological phenotype of the animals [44]. The experiments at the University of Aberdeen were carried out following Home Office guidelines under project license P86A7C9D4.

Male pups were derived from time-mated C57B/6 mothers and cross-fostered onto wild-type or α-casein deficient CD1 mothers. After weaning, the mice were kept on a chow diet for 4 weeks and then on a synthetic diet (D12450B, Research Diets, New Brunswick, NJ, USA) for 8 weeks. The mice were group-housed in groups of 9 for 4 weeks on the chow diet and the first 5 weeks on the synthetic diet. For the last 3 weeks on the synthetic diet, the mice were single-housed to allow an accurate analysis of food intake per mouse. After one week of individual housing, mice were assessed for glucose tolerance using an intraperitoneal glucose tolerance test (IPGTT). The IPGTT (*n* = 9) was carried out after fasting for 5 h. A blood sample (0 min) was taken prior to the intraperitoneal IP glucose injection (1.5 mg/g body weight). This value is reported as fasted glucose. Subsequent blood samples were taken from the tail vein at 15 min, 30 min, 60 min and 120 min and measured using an Accuchek Aviva blood glucose monitor (Roche Diagnostics). The area under the curve (AUC) was calculated by using an Excel add-in (developed by Dr Joel Usansky, University of Irvine, CA, USA).

Mice were sacrificed by euthatal (200 mg/ml) injection (10 µl per g body weight). Serum and tissues were removed for analysis. Body composition was recorded in an ECHO MRI scanner three times during the experimental period: at weaning (3 weeks of age), at 7 weeks of age and at 11 weeks of age.

### Histochemistry

Epididymal white adipose tissue (eWAT) was equilibrated in neutral buffered formalin for 48 h and then stored in 70% ethanol at 4 °C. The tissues were transferred into plastic cassette holders and dehydrated by incubation with 70%, 80%, 95% and 4 × 100% ethanol for 1 h each. The samples were subsequently incubated for 1.5 h in Histoclear for 2 h and then embedded in paraffin wax using a Leica Histo-Embedder and stored at room temperature. Embedded tissue was cut as 5-μm-thick sections using a rotary microtome (Leica RM2135) and transferred to polysine-treated microscope slides and air-dried. Prior to staining, the sections were dewaxed in Histoclear for 5 min, then rehydrated by incubation in 100%, 95% and 70% ethanol for 2 min each.

Sections were then rinsed in water and stained in Haematoxylin Z (CellPath Ltd, RBA-4201-00A) for 5 min, washed in water and incubated in 1% HCl in 70% ethanol for 10 s. Subsequently, sections were rinsed in Scotts water (3.5 g/L NaHCO_3_ and 10 g anhydrous MgSO_4_) for 1 min, rinsed in tap water, stained in Eosin Y (CellPath Ltd, RBB0100 00A) for 30 s and rinsed again in tap water for 30 s. The tissue was dehydrated in 70%, 95% and 100% ethanol for 2 min each and then incubated in Xylene for 10 min. Sections were then mounted on Pertex and photographed using an inverted microscope (Olympus 1X50) at × 100 magnification in bright field (BF) mode. Photographs were taken using the Olympus Cell B software and stored as TIF files. Three sections from three mice per group were analysed for the number, area and diameter of adipocytes using the Fiji Adiposoft (1.13) software.

### DNA analysis

PCR amplifications were carried out using Taq Polymerase from various suppliers. Oligonucleotides were designed using Primer BLAST and the Clone Manager Suite (SciEd Central) and purchased from Invitrogen or Sigma. The modification of the α-casein gene was assessed using the primer pair acas1 (5’ ATG AAA CTC CTC ATC CTC ACC TGC 3’), acas7 (5’ TAG GGG GCA AAA ATG TGT ATT ATC CTA GC 3’) and PGK5 (5’ AAG CGC ATG CTC CAG ACT GCC TTG GGA AA 3’). The PCR was carried out at an annealing temperature of 51 °C. The PCR product generated with the primer pair acas1/acas7 of 688 bp indicates the unmodified α-casein gene. The PCR product generated with the primer pair PGK5/acas7 of 450 bp indicates the targeted α-casein gene. Southern blot analysis was carried out as described [44].

### Biochemical analysis

Liver and tissue lipids were analysed for both total and unesterified fatty acids. Total fat was extracted from liver lobes based on the method by Folch et al. [23], and the weight of fat per microgram of liver tissue was calculated. Total fat was analysed for total fatty acid composition by gas chromatography as described [21]. Measurement of plasma hormones was carried out using Luminex’s xMAP Technology and Milliplex MAP mouse serum adipokine antibodies for insulin and leptin. Measurement of plasma lipids was carried out on terminal blood samples using a Thermo Konelab 30 clinical analyser and kits for HDL and LDL cholesterol and (Microgenics GmbH) and non-esterified fatty acid (NEFA) (Alpha Laboratories).

Milk was isolated at peak lactation (day 10) after cervical dislocation of the mice and processed as described previously [46]. Protein samples were stored at − 80 °C. Milk samples were separated on a 10% polyacrylamide gel and stained with Coomassie Blue.

### Microarray analysis and qPCR

RNA was isolated from liver tissue of offspring nursed by α-casein deficient dams and offspring nursed by wild-type dams at day 21 and day 100 post-partum. Day 100 samples were collected on days 115, 116 and 117 post-partum. To minimise the use of animals, the day 21 (weaning) samples were derived from female offspring, which were not used for further experimentation. Day 100 samples were derived from male mice. Since mice had not entered puberty at weaning (= day 21), the differences between male and female mice were not seen as critical for gene expression at this stage.

Microarray analysis was carried out largely as described [34]. Briefly, total RNA was extracted from 50 mg of frozen liver tissue using an RNeasy Mini Kit with on-column DNAse treatment (Qiagen). The tissue was disrupted in a Precellys homogeniser using 0.4 g of ceramic beads in a volume of 600 ul of buffer RTL. Samples were homogenised once for 10 s and then put on ice for 15 min before being processed as recommended by the kit supplier. RNA quality was measured using a Bioanalyzer 2100 (Agilent Technologies). RNA samples with an RNA integrity number (RIN) above eight were used for analysis. For microarray (Agilent-028005 Whole Genome Mouse 8 × 60 k Array, Agilent Technologies), samples (*n* = 4) derived from liver of mice at day 21 (at the point of weaning) and day 100 from offspring nursed by α-casein deficient dams or wild-type dams (4 experimental groups in total); 100 ng of total RNA was labelled using One Colour Low Input Quick Amp Labelling kit (5190–2305, Agilent Technologies). RNA was amplified in two steps to cRNA using a Cy-3 dye and was purified with RNEasy mini spin columns (Qiagen). For hybridisation, the Gene Expression Hybridisation Kit (Agilent Technologies) was used. Labelled cRNA was hybridised to SurePrint Whole Genome Mouse 8 × 60 k Array microarray slides (Agilent Technologies) rotating at 65 °C for 17 h. The arrays were washed using the Gene Expression Wash Buffer Kit (Agilent Technologies) and then scanned on a SureScan High Resolution Scanner (Agilent Technologies). Microarray data were confirmed by qPCR using the primer combinations listed in Supplementary Table 1 following a method previously described [47].

### Data analysis

The microarray data was analysed using the Bioconductor library limma within R. Loess normalisation was used to remove intensity-dependent dye effects and also to transform intensities to the log2-scale. Replicates for each clone were averaged to obtain one log-ratio value per clone per array. Spots that were deemed to have low quality as judged by the image analysis software were omitted in this averaging process. The moderated *t*-test offered by the limma library was used to calculate *P*-values. Clones were identified as differentially expressed if their fold change expression was equal to or greater than two (up or downregulation) with a significance threshold set at *p* < 0.05. Pathway analysis was carried out using several pathways analysis packages, including EnrichrKG [22], ShinyGO [25], G:Profiler [59] and Metascape [85].

## Results

### α-casein deficiency reduces milk protein concentration and reduces pup growth during lactation

Caseins are the major milk proteins and constitute up to 80% of total milk protein in cattle [61]. In mice, casein proteins account for around 50 mg/ml of milk protein in a total of 100 mg/ml of milk protein [44]. The casein genes are clustered on chromosome 5 in mice (chromosome 6 in cattle, chromosome 4 in humans) (Supplementary Fig. S1). Alpha casein (CSN1S1, CSNA) is the major milk protein in mice [44, 46]. Its inactivation in mice (Supplementary Fig. S1) leads to a reduction of overall milk protein concentration by around 50% (Fig. 1A).

Independent litters of transgenic mice were bred in three groups (Supplementary Table ST2 and Fig. S2). The first group consisted of 3 litters of wild-type control mice nursing their own offspring ([+ / +]WT). The second group consisted of 3 litters of wild-type mice nursing (cross-fostering) offspring of α-casein deficient dams ([+ / +]H). The third group consisted of 3 litters of α-casein deficient dams nursing (cross-fostering) offspring of wild-type dams ([-/-]WT).

Offspring nursed by α-casein deficient dams showed a reduced weight gain during lactation relative to offspring nursed by wild-type dams (Fig. 1B; Supplementary Fig. S3). The difference in weight gain is significant from day 7 of lactation; α-casein deficient dams showed an increased weight gain during the second half of lactation relative to wild-type dams. This difference becomes significant on day 21 of lactation (Fig. 1C, Supplementary Fig. S4). The increased weight gain in α-casein deficient dams may be due to less energy being required for milk production.

In the experimental set-up, litter size was not normalised. Therefore, the effect of litter size and α-casein deficiency could be compared for their relative effects on pup weight gain. Variation in litter size influences pup weight development (e.g. see the data in Supplementary Fig. S3b). However, the effect of litter size on pup (and dam) weight development was much smaller than the effect of α-casein deficiency. We also compared the effect of α-casein deficiency on pup weight gain with data available in the literature; α-casein deficiency has a more pronounced effect on pup growth during lactation than modification of maternal dietary protein (reduced from 20% protein to 8% protein) [57] (Supplementary Fig. S5a) or litter size modulation (Supplementary Fig. S5b) [42].

The reduction in pup weight gain in offspring nursed by α-casein dams becomes more substantial (relative to controls) as lactation progresses (Supplementary Fig. S6a). The correlation follows a logarithmic curve (S6a; *R*^2^ = 0.982). While litter size per se did not have a major impact on weight development of pups in our experimental set-up, larger litters showed a higher degree of weight variation (indicated by the coefficient of variation: CV) in offspring nursed by α-casein deficient dams as well as controls (Supplementary Fig. S6b,c).

Mice from litters nursed by α-casein deficient dams or control dams were analysed for organ weight at day 15 and day 21 of lactation (*n* = 5). Unsurprisingly, absolute organ weight was significantly reduced for all tissues in offspring nursed by α-casein deficient dams at these time points (Supplementary Fig. S7c,e). Relative to overall body weight, however, brain weight was significantly increased (more than two-fold) at both time points (Fig. 1E,F). This is consistent with the observed body morphology displayed (Fig. 1B for day 15 of lactation). Liver weight relative to body weight was significantly reduced at day 15 (Fig. 1E) but was not significantly changed at day 21 (Fig. 1F). Relative to overall body weight, spleen weight was significantly decreased in offspring nursed by α-casein deficient dams at day 21 (Fig. 1F). Relative to overall body weight, kidney and caecum weights were unaffected by the genotype of the dam (Fig. 1E,F).

### Maternal α-casein deficiency extends lifespan in offspring

Offspring, which were nursed by α-casein deficient dams, retained a lower body weight throughout life (Fig. 2A, Supplementary Fig. S7a,b). The difference in body weight was significant on day 21 (weaning), day 177 and day 530 (*p* < 0.001) (Fig. 2B). On day 21, offspring nursed by α-casein deficient dams reached 27% of the weight of offspring nursed by wild-type dams. This rose to 64% on day 171 and to 75% on day 530 (Fig. 2B).

The lifespan of the animals in the three experimental groups was monitored and analysed (Fig. 2D–F). No difference in lifespan was detected between wild-type offspring nursed by their own mothers compared to heterozygous animals nursed by wild-type mothers, while the lifespan of animals nursed by α-casein deficient dams was extended (Fig. 2D). A chi-squared χ^2^ analysis using a log-rank Mantel–Cox test in Graph-Pad Prism and R demonstrated significant extension of lifespan for offspring nursed by α-casein deficient dams (Fig. 2D–F, Table 1), relative to both, wild-type offspring nursed by their own (wild-type) mothers (lifespan extended by 20%, χ^2^ = 8.5 and *p* = 0.004) and heterozygous offspring cross-fostered by wild-type mothers (+ 20%, χ^2^ = 10.6 and *p* = 0.001). Both comparisons show significant changes for female mice (+ 23%, χ^2^ = 5.4 and *p* = 0.02 for wild-type offspring nursed by their own mothers and + 7%, χ^2^ = 5.2 and *p* = 0.02 for heterozygous offspring cross-fostered by wild-type mothers). For male mice, only the lifespan comparison between offspring cross-fostered by α-casein deficient dams and heterozygous offspring cross-fostered by wild-type dams reaches significance (+ 16%, χ^2^ = 7.53 and *p* = 0.006). The overall comparisons for all mice (*p* = 0.007), female mice (*p* = 0.048) and male mice (*p* = 0.048) were significant (Table 1). The Kaplan–Meier plots for this analysis are shown in Fig. 2 for all mice (Fig. 2D), female mice (Fig. 2E) and male mice (Fig. 2F). A one-way ANOVA analysis with Bonferroni post-hoc correction in Graph-Pad and Excel confirmed the findings (Supplementary Fig. S8 and Table ST3).

**Table 1:** Statistical analysis of survival data using a chi-square test (using the log-rank Mantel–Cox test in Graph-Pad and R)

Log-rank Mantel–Cox test							
	Dam	Offspring	Vs	Dam	Offspring	χ^2^	*p*
All	[− / −]	[+ / +]		[+ / +]	[+ / +]	8.479	0.0036
	[+ / +]	[+ / −]		[+ / +]	[+ / +]	0.18	0.67
	[− / −]	[+ / +]		[+ / +]	[+ / −]	10.63	0.0011
Overall comparison						10	0.007
Male	[− / −]	[+ / +]		[+ / +]	[+ / +]	2.7	0.102
	[+ / +]	[+ / −]		[+ / +]	[+ / +]	1.9	0.171
	[− / −]	[+ / +]		[+ / +]	[+ / −]	7.53	0.006
Overall comparison						6.1	0.048
Female	[− / −]	[+ / +]		[+ / +]	[+ / +]	5.41	0.02
	[+ / +]	[+ / −]		[+ / +]	[+ / +]	1	0.316
	[− / −]	[+ / +]		[+ / +]	[+ / −]	5.17	0.023
Overall comparison						6.1	0.048

The individual comparisons between the experimental groups for all mice, male mice and female mice are shown. The overall comparisons for all mice, male mice and female mice are also shown

### Maternal α-casein deficiency programmes body composition and lipid handling in adult animals

In order to study the biological effects of early nutrition on the physiological fate of mice in adulthood, the α-casein mutation was bred onto a CD1 background. At weaning (day 21 post-partum), nine male pups from the middle of the weight distribution (and derived from a total of 6 litters) were combined for the two experimental cohorts (nursed by α-casein deficient [-/-] dams or control [+ / +] dams). They were group-housed on a chow diet up to the age of 53 days post-partum. The mice were then switched to a defined synthetic diet and maintained as a group on that diet for 40 days. Subsequently, the mice were distributed to individual housing cages for 20 days. The weight development of the pups confirmed the observation of Fig. 1C and Supplementary Fig. S7a (when offspring were nursed by C67B/6 dams). The growth of pups nursed by α-casein deficient CD1 dams lagged behind that of control pups nursed by wild-type CD1 dams (supplementary Fig. S9a). There was a period of catch-up growth during adolescence (in which the animals were maintained on a chow diet) (Supplementary Fig. S9b) similar to the one observed in the C57B/6 dams (Supplementary Fig. S7a). After that, the difference in body weight between offspring nursed by α-casein deficient mothers and control mothers was stably maintained in adulthood (Supplementary Fig. S9c,d).

Body composition of offspring nursed by α-casein deficient dams and control dams was analysed on day 21, day 53 and day 80 post-partum using ECHO MRI scanning (Fig. 3A). At all three time points, the percentage of body fat was significantly (*p* < 0.001) reduced in offspring nursed by α-casein deficient dams (relative to controls). At day 15 post-partum, no epididymal adipose tissue was visible in offspring nursed by α-casein deficient dams, whereas it was readily visible in control pups.

Food intake of offspring nursed by α-casein deficient dams or control dams was assessed when the mice were fed a defined synthetic diet. Food intake was measured twice a week by weighing the food consumed during the defined period. For group-housed animals, intake was measured for the whole group and divided by the number of mice and the number of days. Data were averaged over the last 2 weeks of housing (four readings) (Fig. 3B – group). For the individual housing phase, the food intake of all mice (*n* = 9 in each group) was measured for the last 2 weeks of housing (four readings), and the average intake is shown (Fig. 3B – single). Food intake per mouse was between 2 and 2.5 g of diet per day. No significant differences were detected between offspring nursed by α-casein deficient dams and offspring nursed by control dams (Fig. 3B).

Several tissues were isolated from the experimental animals on day 100 post-partum. Histochemical analysis showed a significantly reduced area (*p* < 0.001) and diameter (*p* < 0.01) of adipocytes in epididymal white adipose tissue (eWAT) derived from offspring nursed by α-casein deficient dams relative to controls (Fig. 3C–E). Organ weight analysis (*n* = 9) demonstrated that the absolute weight of the liver and brain is reduced (*p* < 0.05) in offspring nursed by α-casein deficient dams. However, no significant changes in organ weight relative to overall body weight were observed in the liver, heart, kidney or brain (Fig. 4). The absolute weight of epididymal white adipose tissue (eWAT) and brown adipose tissue (BAT) was reduced in offspring nursed by α-casein deficient dams (Fig. 4I,J). The weight of eWAT and BAT was also significantly reduced relative to overall body weight (*p* < 0.05), indicating a reduced investment of energy into adipose depots (Fig. 4K,L). This is associated with a reduction of serum leptin (*p* < 0.001) to 25% of control levels (Fig. 4M). In contrast, serum insulin, fasted glucose and glucose tolerance (as measured by the area under the curve in an intraperitoneal glucose tolerance test) are unaffected by the genotype of the dam (Fig. 4N–P).

Liver tissue derived from offspring nursed by α-casein deficient dams (at day 100) showed a significant reduction in lipid content (*p* < 0.01) relative to controls (Fig. 5A). This was accompanied by a difference in the composition of liver lipids (Fig. 5B). The concentration of mono-unsaturated fatty acids (MUFA) was reduced in the liver derived from offspring nursed by α-casein deficient dams, whereas the concentration of both saturated (SAT) and poly-unsaturated fatty acids (PUFA) was increased (*p* < 0.001). No significant differences in the concentration of serum lipids were observed between the two experimental groups (Fig. 5C).

### Maternal α-casein deficiency programmes liver gene expression in offspring

In order to analyse the molecular changes associated with the increase in lifespan and changes in lipid metabolism, the expression of key genes was analysed. At weaning (day 21), expression of the IGF1 gene was significantly reduced (*p* < 0.001) in the liver of offspring nursed by α-casein deficient dams (relative to control animals) (Fig. 5D). Expression of growth hormone in the pituitary gland of offspring nursed by α-casein deficient dams was also significantly reduced (Fig. 5E). The expression of cell stress genes is often upregulated in long-lived animals [72]. Therefore, liver samples (of animals at day 21) were also assessed for expression of the stress response genes HMOX1 and NQO1. While expression of HMOX1 was significantly upregulated in mice nursed by α-casein deficient dams (Fig. 5G), NQO1 was downregulated (Fig. 5F).

In order to capture the entire transcriptome changes induced in offspring nursed by α-casein deficient dams (relative to offspring nursed by wild-type dams), RNA was isolated from liver tissue of both experimental groups at day 21 and day 100 and analysed by microarray. The microarray data were examined by several quality control analyses, e.g. box plots for data distribution (Supplementary Fig. S10a) and principal component analysis (Supplementary Fig. S10b). The principal component analysis demonstrates that age is the dominant component, which separates the data, followed by the impact of early life nutrition.

The gene expression changes were filtered for changes which occurred with a significance level of *p* < 0.05 and a change of more than two-fold. This identified 385 genes which were upregulated and 243 genes which were downregulated in offspring nursed by α-casein deficient dams (relative to controls) at day 21 (Supplementary Table ST4). At day 100, 119 genes were upregulated, and 98 genes were downregulated in offspring nursed by α-casein deficient dams (relative to controls) (ST4).

In general, the gene expression changes seen at day 21 are more pronounced (maximum upregulation 29-fold; maximum downregulation 84-fold) than the ones seen at day 100 (maximum upregulation 9.8-fold; maximum downregulation 6.6-fold). Moreover, the significance levels of the gene expression changes are more pronounced at day 21 (*p*-values down to 10^−12^) compared to day 100 (*p*-values down to 10^−6^) (Supplementary Fig. S11a–d). This is also reflected in pathway enrichment analyses, which reach a *p*-value of 10^−52^ for the genes upregulated on day 21, but only 10^−8^ for genes upregulated on day 100 (Supplementary Fig. S11e,f).

A list of the top 50 genes whose expression was changed is shown for the four experimental groups (day 21 offspring nursed by wild-type dams, day 21 offspring nursed by α-casein deficient dams, day 100 offspring nursed by wild-type dams, day 100 offspring nursed by α-casein deficient dams) is shown in Supplementary Tables ST5–ST8.

The main upregulated gene in the liver of offspring nursed by α-casein deficient dams was the S100 calcium-binding protein G (Calbindin-D9k) (Supplementary Table ST5). This may be due to the reduced amount of calcium being transported to the offspring via α-casein deficient milk [46]. S100 proteins are a gene family with several dozen members [19]. The S100g gene is mainly expressed in the small intestine and only expressed at very low levels in the liver. However, in response to α-casein deficiency in the lactating mother, expression of the S100g gene was increased 30-fold. At day 100, expression of the S100g returned to very low levels.

The other major upregulated gene families at day 21 and day 100 included glutathione S-transferase genes GSTa1, 2 and 5 (activated between 10- and 20-fold at day 21) (Supplementary Fig. S12a). These genes form part of the cell stress response and have been shown to be activated in numerous other long-lived animal models [72]. Their expression may be a main contributor to the longevity phenotype. The increased expression of GST genes was maintained into adulthood, as GST genes were still significantly increased in the liver of offspring nursed by α-casein deficient dams at day 100 (Supplementary Table ST7 and Fig. S12a).

Other stress response genes, including cytochrome P450 and aldo–keto reductase genes, were also activated in response to the exposure to α-casein deficient milk on day 21. One other notable gene, which was highly activated, was alpha-fetoprotein, a marker of foetal liver, indicating reduced maturation of liver tissue [4]. Similarly, the expression of the non-coding RNA H19 was activated in response to α-casein deficient milk (Supplementary Fig. S12b). H19, like alpha-fetoprotein, is highly expressed in the foetal liver [82] and downregulated after birth. Both alpha-fetoprotein and H19 expression are potent tumour markers in adult liver tissue. In addition, the expression of IGF binding protein 1, a protein inhibiting the effect of IGF1, was increased 22-fold. This increase is also found in mice carrying mutations in the IGF1-growth hormone axis [12] (Fig. 6).

The main downregulated genes at day 21 were the major urinary proteins (MUP) (Supplementary Fig. S13a,b and Table ST6). MUP genes are clustered on mouse chromosome 4 and are an indicator of maturity. They are also reduced in expression in many other long-lived animals, including dwarf mice [12]. Some MUP genes remain downregulated in offspring nursed by α-casein deficient dams at day 100. The pathway analysis software ShinyGO identified the MUP gene locus as a chromosome location, which is significantly enriched in the genes, which were downregulated in offspring nursed by α-casein deficient dams relative to control mice (Supplementary Fig. S14). No gene loci were identified for the other three gene lists: day 21 upregulated, day 100 upregulated and day 100 downregulated.

The most downregulated gene apart from MUP and serpin genes (Supplementary Fig. SF13c) in the liver of offspring nursed by α-casein deficient dams was hydroxy-delta-5-steroid dehydrogenase/3 beta- and steroid delta-isomerase 5 (Hsd3b5). HSD3b5 is a key enzyme in the synthesis of steroid hormones. It is consistently downregulated in long-lived mouse models [75]. HSD3b5 downregulation is also observed in animals exposed to a diet enriched in trans-10, cis-12-conjugated linoleic acid (CLA), which promotes fatty liver in mice [30]. At day 100, HSD3b5 was the most downregulated gene (− 6.6-fold) in mice which had been nursed by α-casein deficient dams.

One group of genes, which was significantly upregulated in mice which were nursed by α-casein deficient dams (relative to control mice) at day 100, are cytochrome P450 genes (Supplementary Fig. S15b). Intriguingly, none of the 6 CYP genes upregulated at day 100 were significantly changed between mice nursed by α-casein deficient dams and control mice at day 21.

The expression data obtained from the microarray were validated for several genes. The representative Egr1 gene (upregulated on day 21 and day 100 in mice nursed by α-casein deficient dams) and Mup19 gene (downregulated on day 21 and day 100 in mice nursed by α-casein deficient dams) were assessed for expression using qPCR (*n* = 6) (Supplementary Fig. S16). The qPCR data confirm the microarray data. Other genes whose expression in the microarray was confirmed by qPCR include Serpin7a, Elovl5, Insig2 and HSD3b5 (Supplementary Fig. S17).

### Pathway analysis indicates a lifelong impact of postnatal nutrition on lipid metabolism and circadian rhythms

The genes, which were changed by more than two-fold with a significance of *p* < 0.05, were analysed using several pathways analysis packages, including EnrichrKG, ShinyGO, G:Profiler and Metascape. Pathways, which were significantly enriched in the gene lists derived from the comparison of mice nursed by α-casein deficient dams and control mice, are shown in Supplementary Tables ST9–ST12. The tables show the top 20 enriched pathways in EnrichrKG (using the KEGG 2021 pathways, LINCS L1000 CRISPR, LINCS L1000 Chemical Perturbation, GO Biological Process 2021 and MGI Mammalian Phenotype 2021 databases). The Gene Ontology and KEGG pathways significantly enriched for all four conditions are summarised in Table 2. A circos plot analysis (carried out in Metascape) demonstrates that there is some overlap in the genes which are upregulated at day 21 and day 100 and some overlap in the genes which are downregulated at both time points (Supplementary Fig. SF18a). An enrichment network cluster analysis of the gene lists derived from the four experimental groups shows that a network of genes upregulated on day 21 forms a cluster distinct from the genes regulated in the other experimental conditions (Supplementary Fig. S15c).

**Table 2:** Top regulated Gene Ontology (GO) and KEGG pathways in mice nursed by α-casein deficient dams and control dams on day 21 and day 100 of life as identified by EnrichrKG and ShinyGo. KEGG and Gene Ontology pathway IDs are shown alongside the p-values of their enrichment

**Day 21 upregulated**		
**term**	**library**	***p*** **-value**
microtubule cytoskeleton organization involved in mitosis	GO:1902850	1.16E-16
mitotic spindle organization	GO:0007052	1.16E-13
DNA metabolic process	GO:0006259	5.28E-11
DNA replication	GO:0006260	1.13E-09
DNA strand elongation involved in DNA replication	GO:0006271	1.67E-08
Glutathione metabolism	KEGG mmu00480	7.12E-08
Metabolism of xenobiotics by cytochrome P450	KEGG mmu00980	1.24E-07
DNA replication	KEGG mmu03030	2.16E-07
Fluid shear stress and atherosclerosis	KEGG mmu05418	0.000002
**Day 21 downregulated**		
**term**	**library**	***p*** **-value**
Peptidyl-tyrosine phosphorylation	GO:0018108	0.000145
Synthesis and degradation of ketone bodies	KEGG mmu00072	0.000172
Enzyme linked receptor protein signaling pathway	GO:0007167	0.000227
Peptidyl-tyrosine modification	GO:0018212	0.000466
Steroid biosynthesis	KEGG mmu00100	0.001499
Ovarian steroidogenesis	KEGG mmu04913	0.002833
Complement and coagulation cascades	KEGG mmu04610	0.003051
**Day 100 upregulated**		
**term**	**library**	***p*** **-value**
Positive regulation of low-density lipoprotein receptor activity	GO:1905599	0.000319
Fatty acid elongation	KEGG mmu00062	0.000477
Biosynthesis of unsaturated fatty acids	KEGG mmu01040	0.000477
Negative regulation of neuron apoptotic process	GO:0043524	0.000725
Positive regulation of neurogenesis	GO:0050769	0.000764
Stress-induced premature senescence	GO:0090400	0.000869
Very long-chain fatty acid metabolic process	GO:0000038	0.000882
PI3K-Akt signaling pathway	KEGG mmu04151	0.00419
MAPK signaling pathway	KEGG mmu04010	0.006805
Homologous recombination	KEGG mmu03440	0.02285
**Day 100 downregulated**		
**term**	**library**	***p*** **-value**
Entrainment of circadian clock by photoperiod	GO:0043153	6.22E-06
Photoperiodism	GO:0009648	7.23E-06
Cellular response to hexose stimulus	GO:0071331	0.000189
Circadian rhythm	KEGG mmu04710	0.000362
Androgen biosynthetic process	GO:0006702	0.000706
Positive regulation of cell activation	GO:0050867	0.00088
Circadian entrainment	KEGG mmu04713	0.009581
Hepatitis B	KEGG mmu05161	0.03674

The pathways altered by reduced protein supply to the offspring nursed by α-casein deficient dams at day 21 are related to growth (e.g. cell cycle, DNA replication; ST9), xenobiotic metabolism (e.g. glutathione metabolism, metabolism of xenobiotics by cytochrome P450; ST9) ketone body synthesis (ST10) and steroid hormone metabolism (ST10).

Some gene expression changes were maintained into adulthood or only became prominent in adulthood (at day 100). E.g., fatty acid elongation and biosynthesis of unsaturated fatty acids were among the significantly upregulated pathways at day 100 (Supplementary Table ST11), suggesting that the lipid composition of liver tissue may be altered as a consequence of nutritional impacts in early postnatal life. This is consistent with the biochemical analysis of liver lipids (Fig. 5A,B). The main pathway downregulated at day 100 was photoperiodism/circadian rhythms, specifically the Periods 1, 2, and 3 (Per1, Per2 and Per3) genes (Supplementary Table ST12). Circadian rhythm regulation is a critical factor in susceptibility to fatty liver (NASH). Inactivation of Per1 results in reduced susceptibility to fatty liver via reducing bile acid synthesis and lipid absorption [26]. This gene expression pattern predicts a reduced susceptibility to fatty liver in offspring nursed by α-casein deficient dams.

### Gene expression patterns in offspring of α-casein deficient mothers are similar to those in dwarf mice and mice exposed to caloric restriction

Mice nursed by α-casein deficient dams share a number of characteristics with dwarf mice. They show reduced adult body weight and an extended lifespan. The genes upregulated and downregulated in Ames and Snell dwarf mice [12] were therefore compared with those upregulated and downregulated in mice nursed by α-casein deficient dams at day 21. The Venn diagram in Supplementary Fig. S19 demonstrates that there is an overlap in regulated genes. Two of the six genes that are shared between the three experimental models are Igfbp1 and Igfbp2. Both of these proteins are able to inhibit IGF action and reduce cell growth [3]. Two of the genes downregulated in all three experimental models are Igf1 and Igfals, which are both stimulators of cell proliferation. The reduction in Igf1 expression was also confirmed by qPCR analysis (Fig. 5D). The expression data derived from the microarray described in this manuscript show that the expression of Igf1 was significantly (*p* < 0.001) reduced on day 21 in offspring nursed by α-casein deficient dams (relative to controls), but that expression was identical to control values at day 100 (Fig. 6A). Similarly, expression of the inhibitory IGF binding protein Igfbp2 was significantly increased at day 21, but not at day 100 (Fig. 6B). Expression of Igfals (an activator of IGF signalling) was significantly reduced (relative to controls) at day 21 but was not significantly different to controls at day 100 (Fig. 6C). In contrast, Igf1 remains significantly reduced in Ames dwarf mice throughout life (Fig. 6D). Expression Igfbp2 is increased in Ames dwarf mice throughout life (Fig. 6E), whereas expression of Igfals is reduced throughout life (Fig. 6F).

Gene expression changes found in the offspring of mice nursed by α-casein deficient dams also overlapped with changes detected in mice undergoing caloric restriction (Supplementary Fig. S20) [73]. This is observed on day 21 and day 100. The overlap is more substantial for genes upregulated (rather than downregulated) by the two interventions (between 10 and 20% of all regulated genes; *p* < 0.05, fold change > 2).

## Discussion

Offspring nursed by α-casein deficient dams showed a significantly increased lifespan (by 20%). The main driver behind the lifespan extension is the reduced growth during lactation. The reduction of body size and its impact on lifespan is entirely due to the genotype of the mother, while the genotype of the offspring is irrelevant to the effect.

The link of reduced growth during early life and an extended lifespan has been shown in other rodent models [24, 39, 56]. Postnatal growth (during lactation) can be reduced by the limitation of dietary protein to the dam (typically a reduction from 20 to 8% of dietary protein) or by an increased litter size. In all three experimental scenarios, reduced growth during the lactation phase resulted in a permanent reduction in adult body weight and an extension of lifespan. This demonstrates that the early postnatal phase is a key determinant of both body size and lifespan. A link between adult body size and lifespan is also observed in other species, notably in dogs, where small breeds have a longer lifespan than large breeds [5]. The link between body height and lifespan is also observed in humans and other primates [16, 67, 83]. This link only holds true within species but, evidently, not across species [71].

The effect sizes of the nutritional intervention on body size and lifespan appear to be linked. The reduction of protein supply to the offspring via the reduced protein content of the maternal diet reduces pup growth during lactation by around 20% (Supplementary Fig. S5a) [57]. This is associated with an increase in lifespan by around 6% (from 765 to 814 days) [56]. In contrast, the growth of pups nursed by α-casein deficient dams is reduced by around 70% at the end of lactation (Supplementary Fig. S5a; Fig. 1B). The lifespan extension of these pups is 22% (from 700 to 863 days) (Fig. 2C). The link between body size and lifespan is also true in the mouse strains carrying a mutation of the growth hormone-IGF axis (Supplementary Fig. S21) [43]. If the lifespan extension of 12 different strains of mice carrying a mutation in the growth hormone-IGF axis (in %) is plotted against the reduction in adult body weight (in %), the trendline suggests a linear and direct correlation (Supplementary Fig. S21). The weight reduction and lifespan extension observed in the offspring of α-casein deficient mice is close to the trendline of that correlation.

Quantity and composition of dietary protein play a key role in metabolic health and have a significant effect on lifespan in many species, including rodents [6, 40, 49, 64]. As a general rule, restriction in protein supply extends lifespan in flies and rodents [6, 27, 70]. The best-characterised example is that of methionine restriction [50], which significantly extends lifespan in mice. Isoleucine restriction has recently been shown to have a similar effect [29]. Intriguingly, shifting the balance of dietary protein in a mouse diet from casein to whey promotes leanness [54]. Our data demonstrate that while the casein concentration in milk is severely reduced in α-casein deficient mice, the concentration of whey proteins is largely unaltered [46]. This suggests that offspring nursed by α-casein deficient dams do not only experience a severe reduction in protein supply but also consume milk with an altered protein composition. Future experiments will address the question of whether protein concentration or composition has a dominant effect in generating the lean phenotype observed. It should be noted that all of the published data demonstrate the effects of long-term dietary interventions in adult animals on lifespan. To our knowledge, the data shown here are the first example assessing a modification of protein composition during early postnatal life on lifespan.

The growth hormone-IGF axis is a well-established regulator of both body size and lifespan [7]. Gene expression in the liver of offspring nursed by α-casein deficient dams at the point of weaning has similarities to gene expression patterns observed in Ames and Snell dwarf mice (and other mouse strains carrying mutations of the growth hormone-IGF axis) [11]. However, many of these gene expression changes are no longer observed at day 100 (Fig. 6). This suggests that the key signals, which are critical for lifespan extension, must be programmed during the early part of life. This is supported by findings in Ames dwarf mice in which growth hormone injections during the first 6 weeks of life substantially reduce lifespan [74]. This establishes the early postnatal period as a phase in which somatotropic signalling is most critical for lifelong biological outcomes. In addition, the gene expression changes observed in offspring nursed by α-casein deficient dams overlap with those seen in mice exposed to caloric restriction, especially with regards to the permanent upregulation of markers of xenobiotic metabolism. This is a feature observed in many long-lived animals [72].

It is not entirely clear why dwarf mice live longer, but the assumption is that dwarf mice with a reduced level of growth hormone-IGF1 axis signalling are less prone to cancer and diabetes [28, 36]. Humans suffering from a genetic growth hormone receptor deficiency (Laron syndrome) also show almost no incidence of diabetes or cancer [8]. However, lifespan is not typically extended in patients with Laron syndrome. This may be due to the fact that growth hormone action is required in humans to maintain cardiovascular health, and humans with growth hormone deficiency (either genetic or after removal of a pituitary tumour) require growth hormone injections [84]. Growth hormone seems to be the key agent of longevity, whereas IGF1 (whose synthesis is driven by growth hormone) seems to be less critical [13, 14]. Nevertheless, inactivation of one copy of the IGF1 receptor is associated with lifespan extension [35]. Inactivation of one copy of the IGF1 receptor exclusively in the brain also extends the average (though not maximum) lifespan. In contrast, if growth hormone is overexpressed in transgenic mice, lifespan is shortened dramatically [9, 81]. This mirrors the effect of acromegaly in humans (increased expression of growth hormone due to excessive activity of the pituitary gland), which also reduces life expectancy [62].

The data shown here are somewhat surprising in that they seem to associate a reduction in growth hormone signalling with a reduction in body fat. Most animals carrying a mutation in the somatotropic axis combine an increased lifespan with an increase in body fat [18]. This is due to the lipolytic activity of growth hormone (therefore, lipolysis is reduced when growth hormone is reduced) [14]. In mice programmed by reduced protein supply during early life, this functional link seems to be changed.

Lipid metabolism has emerged as a potential mediator of lifespan extension in several experimental models [41]. The removal of visceral adipose tissue in rats has been shown to increase lifespan and reduce leptin expression [10, 53]. The deletion of the acyl‐CoA:diacylglycerol acyltransferase 1 (DGAT1) gene in mice leads to animals which are both lean and long-lived. Enhanced β-oxidation of lipids in the liver is a pathway associated with several long-lived mouse models [79]. Moreover, genes involved in somatotropic signalling and lipid biosynthesis are major targets for DNA hypermethylation in long-lived mice [32]. Postnatal catch-up growth (in other words, the reverse of the α-casein deficiency intervention) leads to increased adipose tissue weight and adipocyte size [37]. The reduction in serum leptin observed in offspring nursed by α-casein deficient dams may itself have a critical effect on lifespan. Injection of a leptin antagonist during early postnatal life has been shown to improve metabolic parameters in a mouse model system [17]. Intriguingly, in humans, leanness is mainly associated with extended lifespan in males [77]. However, some experiments in human populations suggest that increased leptin, rather than reduced leptin, is associated with longevity [45].

Taken together, these published data are consistent with the reduction of body fat and a reduction of liver lipid content in offspring nursed by α-casein deficient dams being a critical driver of lifespan extension. The hepatic gene expression data in offspring nursed by α-casein deficient dams identify two major pathways, which are also activated in other experimental systems. The induction of glutathione S-transferase genes found at day 21 and day 100 (Supplementary Fig. S12) is mediated by the Nrf/SKN-1 pathway activated by inhibition of mTORC-1 [63]. mTORC-1 is the mammalian target of rapamycin and mediates the growth-inhibitory effects of rapamycin. Application of rapamycin during early postnatal life has similar biological effects as the reduction of protein supply by the inactivation of the α-casein gene (reduced adult body weight and extended lifespan) [68]. The activation of the FGF21 gene (Supplementary Table ST7) is indicative of the activation of the amino acid-sensitive GCN2-ATF4 signalling pathway [6]. The hepatic gene expression data in offspring nursed by α-casein deficient dams also suggest that photoperiod/circadian rhythms may act as an intermediate between early nutrition and lipid metabolism pathways. Indeed, the relevance of circadian rhythms has emerged as a mediator of nutrition and lifespan [2]. Specifically, the inactivation of the circadian rhythm regulator Per1 in mice prevents obesity on a high-fat diet [26]. This is associated with a reduction in bile acid production via the downregulation of Cyp7A1, the key enzyme determining the synthesis of bile acids in the liver [26]. We observed downregulation of the Per genes and the Cyp7A1 gene in offspring nursed by α-casein deficient dams (relative to control animals) (Supplementary Table ST8).

Body architecture may also be a determinant of lifespan [80]. Body size and lifespan appear to be linked directly in rodents [43]. One potential link between body size and lifespan may be the limited number of available stem cells, which permit tissue repair. Stem cells may be exhausted over the life course. Smaller animals may be smaller, as they contain a smaller number of cells and, therefore, exhaust their stem cell potential and potentially their telomeres less quickly. Extension of telomeres has been shown to affect lifespan and has been suggested as a potential intervention to extend human lifespan [52]. Increasing the number of stem cells using expression vectors for the Yamanaka factors [76] has been shown to extend lifespan in progeroid animal models [69]. Body architecture and the number of available stem cells would provide a simple explanation for the lifespan extension by nutrition in early life. The reduction in protein supply would reduce the number of cell divisions possible during early life and maintain a larger number of stem cells into adulthood.

Intriguingly, different organs respond differently to the reduction in protein supply during lactation. While the growth of the brain appears to be shielded from reduced protein supply, the growth of the spleen is reduced disproportionally (Fig. 1F). The protection of the brain against the effects of reduced protein supply during lactation has been demonstrated in suckling pups raised in extended litters [15]. Caloric restriction has also been shown to affect the growth of different organs differently [20, 51]. Collectively, these data demonstrate that mammals regulate resource distribution to different organs tightly.

All of these mechanisms may contribute to the extended lifespan observed in offspring nursed by α-casein deficient mothers. Many pathways enriched in the offspring of α-casein deficient mice reflect the main changes identified in a large-scale comparison of the transcriptome of long-lived animals (e.g. cellular response to hexose stimulus, complement and coagulation cascades, glutathione metabolism) [78].

In summary, the data shown support several conclusions.

1. Maternal α-casein deficiency substantially restricts the growth of offspring during lactation. This effect is more pronounced than that elicited by other interventions like litter size variation or reducing maternal dietary protein intake. The α-casein deficiency model can, therefore, provide an excellent basis to study the effects of metabolic programming by nutrition in early postnatal life.
2. Maternal α-casein deficiency significantly reduces adult body weight. The body size change is permanent, while the changes in the expression of genes along the somatotropic axis return to control expression levels in adulthood. This underlines the critical importance of early postnatal life for lifelong biological outcomes.
3. Maternal α-casein deficiency programmes lifelong leanness in the offspring. This programming is visible in altered body composition, reduced investment in adipose depots, smaller adipocyte size, reduced liver lipid content and altered liver lipid composition. It is also accompanied by salient changes in liver gene expression relevant to lipid metabolism.
4. Maternal α-casein deficiency significantly increases the lifespan in suckling pups (irrespective of the genotype of the pups). Both reduced body fat and reduced signalling through the somatotropic axis have been suggested as mechanisms which programme lifespan extension. Intriguingly, body size and lifespan extension appear to be linked linearly, irrespective of whether the body size reduction is caused by a nutritional or genetic intervention.

The α-casein deficiency mouse model system provides a basis to study the molecular mechanisms which extend health and lifespan and inform the development of pharmaceutical interventions which achieve similar beneficial health outcomes.

## Supplementary Information

Below is the link to the electronic supplementary material.

Supplementary file1 (DOCX 7703 KB)

Supplementary file2 (DOCX 82 KB)

## Data Availability

The microarray data will be deposited in Gene Expression Omnibus. Other data are available from the authors upon request.
