# Supplementary material for: Maternal α-casein deficiency extends the lifespan of offspring and programmes their body composition
Source: GeroScience. 2024 Jul 12;47(3):3217–39. doi: 10.1007/s11357-024-01273-2 (PMC12181513; doi:10.1007/s11357-024-01273-2)
Supplement: Supplementary file 1 — Supplementary file1 (DOCX 7703 KB) [file 11357_2024_1273_MOESM1_ESM.docx]

**Maternal α-casein deficiency extends the lifespan of offspring and programs their body composition.**

Andreas F. Kolb^1,4^, Claus Mayer^2^, Alina Zitskaja^1^, Linda Petrie^1^, Khulod Hasaballah^1^, Claire Warren^3^, Ailsa Carlisle^3^, Simon Lillico^3^ & Bruce Whitelaw^3^

^1^Nutrition, Obesity and Disease Research Theme, Rowett Institute, University of Aberdeen, UK, ^2^Biomathematics and Statistics Scotland (BioSS), University of Aberdeen,

^3^Roslin Institute, University of Edinburgh, UK

**Supplementary figures**

^4^corresponding author:

Dr Andreas Kolb

Nutrition, Obesity and Disease Research Theme

Rowett Institute

University of Aberdeen

Foresterhill
Aberdeen
AB25 2ZD

UK

phone: 0044-1224-438645

e-mail: [a.kolb@abdn.ac.uk](mailto:a.kolb@abdn.ac.uk)

**Supplementary figures**

**Supplementary figure S1:** Modification of the mouse α-casein (CSN1) gene. **Panel A:** Schematic representation of the mouse casein gene locus. The casein genes are indicated as red arrows. Non-casein genes, which are interspersed between the casein genes are shown as open arrows. The EST evidence of expressed genes is shown below the arrows (indicating the exon/intron structure). Note that EST evidence of expression of the non-casein genes is from non-mammary tissue. **Panel B:** Schematic representation of the mouse α-casein gene (α-cas unmodified), and the α-casein gene modified by homologous recombination (α-cas targeted). The exon-intron structure (exons as boxes, introns as lines) from exon 1 (e1) to exon 34 (e34) is indicated as red boxes. The hygromycin-phosphotransferase-thymidine kinase fusion is represented as a black box. The position of recombinase target sites are indicated as vertical arrows (lox2272, loxP). The position of primer binding sites used for PCR genotyping are indicated as green arrows. The name of the oligonucleotides and the expected product sizes are indicated. The relative positions of the EcoRI restriction sites (EI) and the Southern blot probe (Southern probe) used for Southern blot genotyping (green lines) are indicated as are the sizes of the expected hybridising DNA fragments. **Panel C**: PCR analysis of genomic DNA isolated from the three representative ES cell clones (wild-type [+/+], heterozygous [+/-], and homozygous [-/-] for the modification of the α-casein gene) using the primer combination acas1, acas7, PGK5 (indicative of the 3' end of the α-casein gene after a successful homologous recombination event). A 688bp band represents the unmodified α-casein allele [U]. An α-casein allele modified by homologous recombination is indicated by the occurrence of a 450bp PCR product [T]. **Panel D**: Southern blot analysis of EcoRI digested DNA derived from three representative ES cell clones (wild-type [+/+], heterozygous [+/-], and homozygous [-/-] for the modification of the α-casein gene). The probe indicated in panel B detects a 7.5kb DNA fragment representative of the unmodified α-casein allele [U], a 4.3kb band representative of the targeted α-casein allele [T].

**Supplementary figure S2:** Schematic overview over the cross-fostering experiment. Wildtype mothers and offspring of wildtype mothers are indicated in blue. α-casein deficient mothers and offspring of α-casein deficient mothers (which are heterozygotes) are indicated in red. Sex of the animals is shown (♂, ♀).

**Supplementary figure S3:** Weight gain of pups during the lactation period. **Panel A:** Weight gain of pups (wildtype pups: WT; heterozygous pups of α-casein deficient mothers: H) nursed by α-casein deficient [-/-] and wildtype [+/+] dams (n=3 litters) during the lactation period. Litters were switched after birth. Note that weight gain is determined by the maternal and not by pup genotype. **Panel B:** Weight gain of 3 individual litters nursed by wildtype dams [+/+]. The litters contained 10 (WT10), 11 (WT11), and 13 (WT13) pups. **Panel C:** Weight gain of 3 individual litters nursed by wildtype dams [+/+]. The litters contained 4 (H4), 8 (H8), and 10 (H10) pups. **Panel D:** Weight gain of 3 individual litters nursed by α-casein deficient dams [-/-]. The litters contained 6 (WT6), 8 (WT8), and 11 (WT11) pups. Significance values were determined by one-way ANOVA followed by a Bonferroni post-hoc test in Graph-Pad Prism: ** p<0.01, *** p<0.001.

**Supplementary figure S4:** Weight gain of dams during the lactation period. **Panel A:** Weight gain of α-casein deficient [-/-] and wildtype [+/+] dams nursing wildtype pups (WT) or heterozygous pups derived from α-casein deficient mothers (H) during the lactation period. **Panel B:** Weight gain of 3 individual wildtype dams [+/+] nursing wildtype litters containing 10 (WT10), 11 (WT11), and 13 (WT13) pups. **Panel C:** Weight gain of 3 individual wildtype dams [+/+] nursing α-casein heterozygous litters containing 6 (H4), 8 (H8), and 10 (H10) pups. **Panel D:** Weight gain of 3 individual α-casein deficient dams [-/-] nursing wildtype litters containing 4 (WT4), 8 (WT8), and 11 (WT11) pups. Weights are shown as percentage of the weight at mating. Significance values were determined by one-way ANOVA followed by a Bonferroni post-hoc test in Graph-Pad Prism. ** p<0.01.

**Supplementary figure S5:** Comparison of effect size of α-casein deficiency, litter size and altered maternal diet. **Panel A:** Comparison of weight development of pups in this manuscript (acas [-/-]) and in Ozanne et al. 2004 (8% MD; 8% of protein in maternal diet) during lactation. The data are expressed as % of control pup weight (nursed by wildtype mothers or mothers on a standard diet containing 20% protein). Error bars represent standard deviation (n=24). Male and female mice were used as there is no sexual dichotomy during lactation. **Panel B:** Comparison of weight development of pups in this manuscript and in Kappeler et al. 2009. Kappeler et al. modulate pup growth by altering litter size in SV129/C57B6 hybrid mice. The data for pups grown in litters of 6 and 10 pups were correlated with control litters of 3 pups. Data shown are for male mice only.

**Supplementary figure S6:** Variation in pup weight in α-casein deficient mothers. **Panel A:** Development of pup weight over time. The weight of pups nursed by α-casein deficient mothers was correlated with the weight of pups nursed by control dams over the period of lactation. **Panel B:** Coefficient of variation of pup weight in litters of varying size nursed by wildtype dams. **Panel C:** Coefficient of variation of pup weight in litters of varying size nursed by α-casein deficient dams.

**Supplementary figure S7:** Weight development and lifespan of offspring nursed by wildtype [+/+] and α-casein deficient [-/-] dams. **Panel A:** Average weight of offspring nursed by wildtype [+/+] and α-casein deficient [-/-] dams over the first 60 days of life. Male (M) and female (F) are shown separately. **Panel B:** Average weight of offspring nursed by wildtype [+/+] and α-casein deficient [-/-] dams over the entire lifespan. **Panels C-F:** Organ weight in offspring at day 15 and day 21 post-partum. **Panel C:** Liver, kidney and brain weights in pups nursed by wildtype dams [+/+] or α-casein deficient dams [-/-] at day 15 of lactation (in mg). **Panel D:** Percentage of liver, kidney and brain weights relative to total body weight in pups nursed by wildtype [+/+] and α-casein deficient dams [-/-] dams at day 15 of lactation. **Panel E:** Brain, liver, kidney spleen and caecum weights in pups nursed by wildtype dams [+/+] or α-casein deficient dams [-/-] at day 21 of lactation (weaning). **Panel F:** Percentage of brain, liver, kidney spleen and caecum weights relative to total body weight in pups nursed by wildtype [+/+] and α-casein deficient dams [-/-] dams at day 21 of lactation. Significance values were determined by one-way ANOVA followed by a Bonferroni post-hoc test in Graph-Pad Prism: * p<0.05, *** p<0.001.

**Supplementary figure S8:** ANOVA analysis of the lifespan of offspring nursed by wildtype [+/+] and α-casein deficient [-/-] dams. One-way ANOVA using Bonferroni post-hoc correction data are shown for all (**Panel A**), male (**Panel B**) and female (**Panel C**) mice. The corresponding p-values are shown in supplementary table **ST3**.

**Supplementary figure S9:** Weight development of C57B/6 pups nursed by α-casein deficient [-/-] or wildtype [+/+] CD1 foster mothers. **Panel A:** Weight development during lactation. The data are derived from 6 independent litters with [+/+] mothers nursing 10 pups each, and 6 independent litters with [-/-] mothers nursing 10 pups each. **Panel B:** Weight development in group housed mice on a chow diet (n=9). **Panel C:** Weight development of group housed mice on a synthetic control diet (n=9). **Panel D:** Weight development of individually housed mice on a synthetic control diet (n=9).

**Supplementary figure S10:** Microarray analysis of gene expression in mice nursed by wildtype (WT) or α-casein deficient dams (KO) at day 21 and day 100 post-partum (n=4). **Panel A:** Boxplot analysis of data in the 16 samples. **Panel B:** Principal component analysis of sample in the microarray analysis, including animals nursed by wild-type dams at day 21 (WT21) and day 100 (WT100) and nursed by α-casein deficient dams at day 21 (KO21) and day 100 (KO100). Note that age separates samples more than maternal genotype.

**Supplementary figure S11:** Statistical significance of gene expression and pathway enrichment changes. The p-values for the top 50 regulated genes are shown for day 21 (upregulated genes: **Panel A**; downregulated genes: **Panel B**), and day 100 (upregulated genes: **Panel C**; downregulated genes: **Panel D**). The p-values for the top 20 regulated pathways (identified by Enrichr-KG) are shown for day 21 (upregulated genes: **Panel E**; downregulated genes: **Panel F**), and day 100 (upregulated genes: **Panel G**; downregulated genes: **Panel H**).

**Supplementary figure S12: Panel A:** Expression of 5 glutathione S-transferase genes upregulated in offspring nursed by α-casein deficient dams on day 21. The expression values read from the microarray signal are shown. Error bars represent standard deviation. The 5 GST genes are significantly changed between wildtype dam nursed offspring (WT21) and α-casein deficient dam nursed offspring (KO21) (p<0.001) at day 21. GSTa1, 2 and 5 are also significantly changed between wildtype dam nursed offspring (WT100) and α-casein deficient dam nursed offspring (KO100) at day 100 (p<0.05). **Panel B:** Expression of maternally expressed noncoding RNAs Mirg and H19 (5 different probes) upregulated in α-casein deficient dam nursed mice at day 21 (p<0.01). At day 100 no significant changes between control and offspring nursed by α-casein deficient dams are detected.

**Supplementary figure S13: Panel A:** Expression of 11 major urinary protein (MUP) genes (14 probes) downregulated in offspring nursed by α-casein deficient dams on day 21. The expression values read from the microarray signal are shown. Error bars represent standard deviation. The 11 MUP genes are significantly changed between wildtype dam nursed offspring (WT21) and α-casein deficient dam nursed offspring (KO21) (p<0.001) at day 21. Only MUP 10 is also significantly changed between wildtype dam nursed offspring (WT100) and α-casein deficient dam nursed offspring (KO100) at day 100 (p<0.05). **Panel B:** Expression of 6 MUP pseudogenes (7 probes) downregulated in α-casein deficient dam nursed mice at day 21 (p<0.01). At day 100 no significant changes between control and offspring nursed by α-casein deficient dams are detected. **Panel C:** Expression of 3 Serpin genes (4 probes) downregulated in α-casein deficient dam nursed mice at day 21 (p<0.01). At day 100 no significant changes between control and offspring nursed by α-casein deficient dams are detected.

**Supplementary figure S14:** Genome enrichment plot of genes downregulated in the liver of offspring nursed by α-casein deficient dams (relative to offspring nursed by wildtype dams). The plot was generated using the ShinyGO software and the significantly enriched MUP gene locus on chromosome 4 is indicated in lilac.

**Supplementary figure S15: Panel A:** Expression of 4 glutathione S-transferase genes upregulated in offspring nursed by α-casein deficient dams at day 100. The expression values read from the microarray signal are shown. Error bars represent standard deviation. The 4 GST genes are also significantly changed between wildtype dam nursed offspring (WT21) and α-casein deficient dam nursed offspring (KO21) at day 21 (p<0.05). **Panel B:** Expression of 6 cytochrome P450 genes significantly upregulated in offspring nursed by α-casein deficient dams at day 100 (p<0.01). On day 21 no significant changes between control and offspring nursed by α-casein deficient dams are detected.

**Supplementary figure S16:** qPCR confirmation of microarray results. Expression of genes in RNA derived from liver tissue isolated from experimental animals at day 21 post-partum (i.e., at the end of the lactation period) and at day 100 (at the end of an 8-week diet intervention). **Panel A:** Microarray RNA expression data for the Egr1 gene at age day 21 and day 100 in animals nursed by wildtype (WT) and α-casein deficient dams (KO) (n=4). **Panel B:** Microarray data for the Mup19 gene in the 4 experimental conditions (n=4). **Panel C:** qPCR expression data for the Egr1 gene in the 4 experimental conditions (n=4). **Panel D:** qPCR expression data for the Mup19 gene in the 4 experimental conditions (n=3 for day 21 and n=6 for day 100). Gene expression of genes was correlated with expression of the reference gene β-actin for the qPCR analysis. The comparisons between animals nursed by α-casein deficient mothers (KO) or control mothers (WT) were analysed by ANOVA. p<0.05: *, p<0.01: **, p<0.001: ***.

**Supplementary figure S17:** Expression of genes in RNA derived from liver tissue isolated from experimental animals at day 21 post-partum (i.e., at the end of the lactation period) and at day 100 (at the end of an 8-week diet intervention). Values are derived from the microarray (n=4) for Serpin 7a (**panel A**), Elovl5 (**panel B**), Insig2 (**panel C**) and Hsd3b5 (**panel D**), and from qPCR analysis for Serpin 7a (n=3) (**panel E**), Elovl5 (n=3) (**panel F**), Insig2 (n=3) (**panel G**) and Hsd3b5 (n=6) (**panel H**). The values for the qPCR analysis refer to day 100. Gene expression of genes was correlated with expression of the reference gene β-actin for the qPCR analysis. The comparisons between animals nursed by α-casein deficient mothers (KO) or control mothers (WT) were analysed by ANOVA. p<0.05: *, p<0.01: **, p<0.001: ***.

**Supplementary figure S18:** Gene list analysis of all conditions. **Panel A:** Circos plot of gene list-overlap analysis (generated in Metascape). Comparison of gene expression in liver samples derived from offspring nursed by α-casein deficient dams relative to offspring nursed by wildtype dams. Genes upregulated (d21up) or downregulated (d21down) by more than 2-fold with p<0.05 at day 21. Genes upregulated (d100up) or downregulated (d100down) by more than 2-fold with p<0.05 at day 100. **Panel B:** Circos plot for the same comparisons expanded via shared ontologies. **Panel C:** Enrichment network clusters for the 4 gene lists (generated in Metascape). Note that one network of related genes which is upregulated on day 21 is separate from genes in the other 3 analysis groups.

**Supplementary figure S19**. Comparison of gene expression changes in Ames (4 months of age) and Snell (6 months of age) dwarf mice and offspring nursed by α-casein deficient dams on day 21. **Panel A:** Venn diagram of genes upregulated by more than 2-fold with p<0.05. **Panel B:** Venn diagram of genes downregulated by more than 2-fold with p<0.05. The genes which are common between all 3 experimental groups are shown underneath the Venn diagram.

**Supplementary figure S20:** Venn diagram of comparisons of genes activated by >2 fold with a p-value of <0.05 in mice exposed to caloric restriction (30%) (Suchacki et al. 23; GSE230492, GSE230402) and offspring nursed by α-casein deficient dams at day 21 and day 100 of life. The comparisons are shown for genes upregulated by caloric restriction and genes upregulated at day 21 (**Panel A**), genes upregulated by caloric restriction and genes upregulated at day 100 (**Panel B**), genes downregulated by caloric restriction and genes downregulated at day 21 (**Panel C**), genes downregulated by caloric restriction and genes downregulated at day 100 (**Panel D**).

**Supplementary figure 21:** Lifespan extension correlated with adult body weight. Data derived for 12 different mouse strains carrying gene mutations affecting the growth hormone-IGF axis from Kim & Lee, 2019 (table 1) are shown as blue dots. The data for offspring nursed by α-casein deficient dams are shown as red dots. Weight data are shown for day 177 (-36% of controls) and day 530 (-25% of controls). The data for mice programmed by early nutrition cluster around the trendline of the correlation between body weight reduction and lifespan extension.
