# Supplementary material for: Maternal α-casein deficiency extends the lifespan of offspring and programmes their body composition
Source: GeroScience. 2024 Jul 12;47(3):3217–39. doi: 10.1007/s11357-024-01273-2 (PMC12181513; doi:10.1007/s11357-024-01273-2)
Supplement: Supplementary file 2 — Supplementary file2 (DOCX 82 KB) [file 11357_2024_1273_MOESM2_ESM.docx]

**Maternal α-casein deficiency extends the lifespan of offspring and programs their body composition.**

Andreas F. Kolb^1,4^, Claus Mayer^2^, Alina Zitskaja^1^, Linda Petrie^1^, Khulod Hasaballah^1^, Claire Warren^3^, Ailsa Carlisle^3^, Simon Lillico^3^ & Bruce Whitelaw^3^

^1^Nutrition, Obesity and Disease Research Theme, Rowett Institute, University of Aberdeen, UK, ^2^Biomathematics and Statistics Scotland (BioSS), University of Aberdeen,

^3^Roslin Institute, University of Edinburgh, UK

**Supplementary tables**

^4^corresponding author:

Dr Andreas Kolb

Nutrition, Obesity and Disease Research Theme

Rowett Institute

University of Aberdeen

Foresterhill
Aberdeen
AB25 2ZD

UK

phone: 0044-1224-438645

e-mail: [a.kolb@abdn.ac.uk](mailto:a.kolb@abdn.ac.uk)

**Supplementary tables**

| **gene ID** | **primer name** | **primer sequence** | **annealing temp.** | **product size** | **Genbank** |
| --- | --- | --- | --- | --- | --- |
| m-b-actin | b-actin3 | GAYGAGGCYCAGAGCAAGAGAG | 60°C | 387bp | XM_030254057.1 |
|  | b-actin4 | GTCCCGGCCAGCCAGGTCCAG |  |  |  |
| IGF1 | mIGF1-10 | GCGCTCTGCTTGCTCACCTTCAC | 57°C | 227bp | NM_001314010.1 |
|  | mIGFI-11 | GGGGCACAGTACATCTCCAGT |  |  |  |
| mGH | mGH3 | TGGACAGATCACTGCTTGGC | 55°C | 203bp | NM_008117.3 |
|  | mGH4 | TAGGCACGCTCGAACTCTTT |  |  |  |
| mNQO1 | mNQO1-1 | TAGCCTGTAGCCAGCCCTAA | 58°C | 531bp | NM_008706.5 |
|  | mNQO1-2 | TCTGGAAAGGACCGTTGTCG |  |  |  |
| mHMOX1 | mHMOX1-3 | TGGCCCACGCATATACCCGC | 53°C | 385bp | NM_010442.2 |
|  | mHMOX1-4 | TGGGGTTTCCCTCGGGGTGTC |  |  |  |
| mELOVL5 | mElovl5-1 | TTCCTCTTGCATCGCGGCT | 58°C | 544bp | NM_134255.3 |
|  | mElovl5-2 | ACGGTGATCTGGTGGTTGTT |  |  |  |
| mSerpin A7 | mSerpin A7-1 | GGCTTCTTGGGCATGTGCTATC | 59°C | 310bp | NM_177920.5 |
|  | mSerpin A7-2 | GGGTGCTAGAGCCAGATCCA |  |  |  |
| mInsig2 | mINSIG2-1 | TTGGGAAGAGGAGCTGTTTATG | 58°C | 129bp | NM_001271531.1 |
|  | mINSIG2-2 | GAACTCTCTCCAGGTAAGCCG |  |  |  |
| mMUP19 | mMUP19-1 | TGCTGTTGCTGCTGTGTTTG | 60°C | 395bp | XM_006537526.5 |
|  | mMUP19-2 | CAGCTGGAAGGTTTCCCCAT |  |  |  |
| mHSD3b5 | mHSD3b5-1 | CATCCAGATCAAGAAGGGAACTG | 54°C | 513bp | NM_008295.2 |
|  | mHSD3b5-2 | AGGAATTTGGTCCAGCCACA |  |  |  |

**Supplementary table ST1:** Oligonucleotides for gene expression analysis by qPCR. The primer names and sequences are shown. The annealing temperatures for the PCRs, the expected product sizes of the PCR products, and the Genbank accession numbers for mRNAs detected by the primer pairs are also shown.

| **dam** | **dam GT** | **litter size** | **mother of litter** | **pup GT** | **designation** |
| --- | --- | --- | --- | --- | --- |
| #10 | [+/+] | 13 | #10 | WT | [+/+] WT13 |
| #8 | [+/+] | 11 | #8 | WT | [+/+] WT11 |
| #6 | [+/+] | 10 | #6 | WT | [+/+] WT10 |
| #7 | [+/+] | 4 | #5 | H | [+/+] H4 |
| #12 | [+/+] | 8 | #2 | H | [+/+] H8 |
| #13 | [+/+] | 10 | #1 | H | [+/+] H10 |
| #2 | [-/-] | 6 | #12 | WT | [-/-] WT6 |
| #1 | [-/-] | 11 | #13 | WT | [-/-] WT11 |
| #5 | [-/-] | 8 | #7 | WT | [-/-] WT8 |

**Supplementary table ST2:** Tabular view of the experimental set-up. Wildtype pups born to wildtype [+/+] mothers are indicated as WT. Pups born to α-casein deficient [-/-] mothers are heterozygous and are indicated as H.

| **pairwise comparison (Excel)** | | |  |  |  |  |
| --- | --- | --- | --- | --- | --- | --- |
|  | **dam** | **offspring** | **vs** | **dam** | **offspring** | **p** |
| all | [-/-] | [+/+] |  | [+/+] | [+/+] | 0.0004 |
|  | [+/+] | [+/-] |  | [+/+] | [+/+] | 0.595 |
|  | [-/-] | [+/+] |  | [+/+] | [+/-] | 0.004 |
| male | [-/-] | [+/+] |  | [+/+] | [+/+] | 0.039 |
|  | [+/+] | [+/-] |  | [+/+] | [+/+] | 0.171 |
|  | [-/-] | [+/+] |  | [+/+] | [+/-] | 0.02 |
| female | [-/-] | [+/+] |  | [+/+] | [+/+] | 0.0001 |
|  | [+/+] | [+/-] |  | [+/+] | [+/+] | 0.306 |
|  | [-/-] | [+/+] |  | [+/+] | [+/-] | 0.065 |
|  |  |  |  |  |  |  |
| **overall comparison with post-hoc correction (Graph-Pad)** | | | | | |  |
|  | **dam** | **offspring** | **vs** | **dam** | **offspring** | **p** |
| all | [-/-] | [+/+] |  | [+/+] | [+/+] | *** |
|  | [+/+] | [+/-] |  | [+/+] | [+/+] | ns |
|  | [-/-] | [+/+] |  | [+/+] | [+/-] | * |
| overall comparison | |  |  |  |  | 0.0008 |
| male | [-/-] | [+/+] |  | [+/+] | [+/+] | ns |
|  | [+/+] | [+/-] |  | [+/+] | [+/+] | ns |
|  | [-/-] | [+/+] |  | [+/+] | [+/-] | ns |
| overall comparison | |  |  |  |  | 0.041 |
| female | [-/-] | [+/+] |  | [+/+] | [+/+] | ** |
|  | [+/+] | [+/-] |  | [+/+] | [+/+] | ns |
|  | [-/-] | [+/+] |  | [+/+] | [+/-] | ns |
| overall comparison | |  |  |  |  | 0.0083 |

**Supplementary table ST3**: Statistical analysis of survival data using one-way ANOVA. The individual comparisons between the experimental groups for all mice, male mice and female mice generated in Excel are shown in the upper half of the table. The overall comparisons for all mice, male mice and female mice generated by one-way ANOVA with Bonferroni post-hoc correction in Graph-Pad Prism are shown in the lower half of the table.

| **treatment** | **probes changed** | **unknown calls** | **multiple calls** | **genes changed** |
| --- | --- | --- | --- | --- |
| day 21 up | 462 | 9 | 69 | 385 |
| day 21 down | 304 | 10 | 61 | 243 |
| day 100 up | 136 | 2 | 15 | 119 |
| day 100 down | 119 | 12 | 9 | 98 |

**Supplementary table ST4:** Probes and gene changes in liver RNA samples derived from offspring nursed by α-casein deficient dams relative to offspring nursed by wild-type control dams. Probes and genes increased (day 21 up) or decreased (day 21 down) in offspring nursed by α-casein deficient dams at day 21 and increased (day 100 up) and decreased (day 100 down) at day 100 are shown. Probes and genes changed by more than two-fold with a significance value of p<0.05 are shown. Note that the gene expression changes are more pronounced on day 21.

| **Agilent probe** | **GeneName** | **Description** | **GenBank** | **fold change d21** | **p d21** | **p d100** |
| --- | --- | --- | --- | --- | --- | --- |
| A_55_P2111163 | S100g | S100 calcium binding protein G (S100g), mRNA | NM_009789 | 29.55 | 1.88E-12 | 0.157037 |
| A_55_P2013236 | S100g | S100 calcium binding protein G (S100g), mRNA | NM_009789 | 26.30 | 6.16E-12 | 0.176042 |
| A_30_P01018914 | Gsta1 | Glutathione S-transferase A1, mRNA | NM_008181.3 | 23.43 | 0.001242 | **0.02070** |
| A_51_P447545 | Igfbp1 | insulin-like growth factor binding protein 1, mRNA | NM_008341 | 21.87 | 0.000456 | 0.957181 |
| A_55_P2032946 | Gsta1 | Glutathione S-transferase A1, mRNA | NM_008181.3 | 21.42 | 0.000485 | **0.02474** |
| A_30_P01028766 | Gsta1 | Glutathione S-transferase A1, mRNA | NM_008181.3 | 18.91 | 0.000786 | **0.02850** |
| A_51_P510891 | Afp | alpha fetoprotein, mRNA | NM_007423 | 15.16 | 9.06E-09 | 0.704355 |
| A_51_P493649 | Sult1e1 | sulfotransferase family 1E, member 1, mRNA | NM_023135 | 15.02 | 9.70E-09 | 0.802996 |
| A_55_P2102065 | Gsta5 | Glutathione S-Transferase Alpha 5, mRNA | NM_001122660.1 | 14.29 | 0.000155 | **0.03368** |
| A_55_P1966749 | Cyp2s1 | cytochrome P450, family 2, subfamily s, polypeptide 1, mRNA | NM_028775 | 12.85 | 0.049762 | 0.929343 |
| A_51_P318830 | Syt10 | synaptotagmin X, mRNA | NM_018803 | 12.61 | 0.000184 | 0.764491 |
| A_52_P445360 | Krt20 | keratin 20, mRNA | NM_018803 | 12.39 | 1.82E-08 | 0.161623 |
| A_55_P2170454 | Gsta2 | glutathione S-transferase, alpha 2 (Yc2), mRNA | NM_008182 | 10.90 | 3.88E-05 | **0.02248** |
| A_55_P2069969 | Gsta5 | Glutathione S-Transferase Alpha 5, mRNA | NM_001122660.1 | 10.69 | 3.47E-06 | **0.02946** |
| A_55_P2079167 | Dnm1 | dynamin 1, mRNA | NM_010065 | 9.99 | 3.30E-05 | 0.919889 |
| A_51_P331288 | Akr1b7 | aldo-keto reductase family 1, member B7, mRNA | NM_009731 | 9.90 | 5.83E-07 | 0.122498 |
| A_55_P2070869 | Lcn2 | lipocalin 2, mRNA | NM_008491 | 9.44 | 2.05E-05 | **0.04166** |
| A_55_P2048867 | Mirg | miRNA containing gene/maternally-expressed gene 9, ncRNA | NR_028265.1 | 9.22 | 4.67E-05 | 0.921839 |
| A_30_P01021588 | H19 | imprinted ncRNA, maternally expressed | NR_130974.1 | 9.19 | 0.023933 | 0.894816 |
| A_52_P367760 | Calml4 | calmodulin-like 4, transcript variant 1, mRNA | NM_138304 | 9.19 | 1.30E-10 | 0.605793 |
| A_30_P01025068 | H19 | imprinted ncRNA, maternally expressed | NR_130974.1 | 8.92 | 0.006208 | 0.406197 |
| A_30_P01026972 | H19 | imprinted ncRNA, maternally expressed | NR_130974.1 | 8.56 | 0.004014 | 0.935516 |
| A_55_P2144686 | Dmbt1 | deleted in malignant brain tumors 1, mRNA | NM_007769 | 8.41 | 0.041615 | 0.932665 |
| A_30_P01028007 | H19 | imprinted ncRNA, maternally expressed | NR_130974.1 | 8.40 | 0.004361 | 0.607269 |
| A_55_P1963017 | Stfa1 | stefin A1, mRNA | NM_001082543 | 8.32 | 6.29E-09 | 0.53384 |
| A_30_P01028827 | H19 | imprinted ncRNA, maternally expressed | NR_130974.1 | 8.17 | 0.0037 | 0.671332 |
| A_30_P01027466 | Cstdc5 | cystatin domain containing 5, mRNA | NM_001082546.1 | 8.12 | 1.21E-08 | 0.453971 |
| A_52_P487686 | Asns | asparagine synthetase, mRNA | NM_012055 | 8.11 | 0.000163 | 0.260877 |
| A_55_P1959748 | Orm3 | orosomucoid 3, mRNA | NM_013623 | 7.88 | 2.63E-06 | 0.362972 |
| A_51_P311958 | Cdk1 | cyclin-dependent kinase 1, mRNA | NM_007659 | 7.58 | 0.011827 | 0.504902 |
| A_55_P2048588 | Lepr | leptin receptor, transcript variant 2, mRNA | NM_010704 | 7.17 | 0.000794 | 0.980726 |
| A_55_P2177911 | Bex1 | brain expressed gene 1, mRNA | NM_009052 | 6.78 | 0.000425 | 0.930942 |
| A_55_P2031021 | Brme1 | Break Repair Meiotic Recombinase Recruitment Factor 1, mRNA | XM_030243821.1 | 6.70 | 5.99E-07 | **0.01769** |
| A_55_P2106241 | Apoa4 | apolipoprotein A-IV, mRNA | NM_007468 | 6.27 | 0.000113 | 0.131214 |
| A_51_P327491 | Mgst2 | microsomal glutathione S-transferase 2, mRNA | NM_174995 | 6.20 | 2.23E-10 | 0.347408 |
| A_51_P150120 | Fbxw15 | F-box and WD-40 domain protein 15, mRNA | NM_199036 | 6.05 | 6.75E-07 | 0.590201 |
| A_55_P2145977 | Tuba8 | tubulin, alpha 8, mRNA | NM_017379 | 5.96 | 0.021772 | 0.826543 |
| A_66_P119518 | Tff3 | trefoil factor 3, intestinal, mRNA | NM_011575 | 5.79 | 0.002666 | 0.12486 |
| A_51_P456208 | Serpina7 | serine (or cysteine) peptidase inhibitor member 7, mRNA | NM_177920 | 5.67 | 3.35E-09 | **1.26E-5** |
| A_51_P162955 | Stfa2l1 | stefin A2 like 1, mRNA | NM_173869 | 5.40 | 4.74E-08 | 0.522957 |
| A_52_P398925 | Igdcc4 | immunoglobulin superfamily, DCC subclass, member 4, mRNA | NM_020043 | 5.32 | 0.004646 | 0.462601 |
| A_66_P114167 | Slc35f2 | solute carrier family 35, member F2, mRNA | NM_028060 | 5.30 | 4.65E-06 | 0.505501 |
| A_55_P1985351 | Cgref1 | cell growth regulator with EF hand domain 1, mRNA | NM_026770 | 5.23 | 4.30E-05 | **0.01668** |
| A_51_P372550 | Tceal5 | transcription elongation factor A (SII)-like 5, mRNA | NM_177919 | 5.18 | 0.017017 | 0.681297 |
| A_66_P104980 | Gal3st1 | galactose-3-O-sulfotransferase 1, mRNA | NM_016922 | 5.01 | 2.86E-06 | **0.03616** |
| A_55_P2013823 | Aqp3 | aquaporin 3, mRNA | NM_016689 | 5.00 | 1.29E-05 | 0.895317 |
| A_51_P245090 | Acmsd | amino carboxymuconate semialdehyde decarboxylase, mRNA | NM_001033041 | 4.87 | 3.90E-05 | 0.432832 |
| A_55_P2161347 | Slc51b | solute carrier family 51, beta subunit, mRNA | NM_178933 | 4.79 | 7.25E-05 | 0.363726 |
| A_52_P229943 | Marco | macrophage receptor with collagenous structure, mRNA | NM_010766 | 4.78 | 0.000757 | 0.807425 |

**Supplementary table ST5:** Top 50 genes **upregulated** in the liver of offspring nursed by α-casein deficient dams relative to offspring nursed by wildtype dams at **day 21** (weaning). Eight of the top 51 genes upregulated are glutathione S-transferase genes (highlighted in blue). Six of the top 50 genes upregulated are maternally expressed non-coding RNAs (5 of which are H19 specific probes). All gene expression changes between WT nursed and KO nursed animals are significant on day 21. 11 of the 50 genes are still changed significantly on day 100 (10 with p<0.05 and 1 with p<0.001). The p-values of the gene expression changes on day 21 (p d21) and day 100 (p d100) are coloured by Microsoft Excel conditional formatting. The lowest p-values are in red, the highest p-values are in green. Genes expressing non-coding RNAs are marked in red in the GenBank column.

| **Agilent probe** | **GeneName** | **Description** | **GenBank** | **fold change d21** | **p d21** | **p d100** |
| --- | --- | --- | --- | --- | --- | --- |
| A_55_P2004606 | Serpina4-ps1 | serine peptidase inhibitor, clade A, member 4, pseudogene 1, non-coding RNA | NR_002861 | -84.41 | 1.82E-06 | 0.113058 |
| A_55_P1974080 | Mup14 | major urinary protein 19, mRNA | NM_001135127 | -73.15 | 1.28E-10 | 0.991549 |
| A_55_P1971237 | Mup3 | major urinary protein 3, mRNA | NM_001039544 | -68.30 | 1.26E-10 | 0.635326 |
| A_55_P1979904 | Mup14 | major urinary protein 9, mRNA | NM_001126319 | -66.50 | 2.52E-10 | 0.652875 |
| A_55_P1987403 | Mup18 | major urinary protein 18, mRNA | NM_001199333 | -64.04 | 1.73E-10 | 0.970663 |
| A_55_P2076196 | Mup11 | major urinary protein 17, mRNA | NM_001200006 | -63.91 | 8.09E-11 | 0.743473 |
| A_55_P2035029 | Mup-ps13 | major urinary protein pseudogene 13, ncRNA | ENSMUSG00000095532 | -60.75 | 1.19E-10 | 0.563017 |
| A_55_P2033321 | Mup-ps6 | major urinary protein pseudogene 6, ncRNA | ENSMUST00000153865 | -59.22 | 1.78E-10 | 0.227725 |
| A_55_P1997003 | Serpina4-ps1 | serine peptidase inhibitor, clade A, member 4, pseudogene 1, non-coding RNA | NR_002861 | -57.63 | 6.20E-06 | 0.118383 |
| A_55_P2111980 | Hsd3b5 | hydroxy-delta-5-steroid dehydrogenase, 3 beta- and steroid delta-isomerase 5 (Hsd3b5), mRNA | NM_008295 | -56.77 | 8.25E-05 | **0.02629** |
| A_52_P412506 | Mup5 | major urinary protein 5, mRNA | NM_008649 | -54.81 | 1.07E-09 | 0.171044 |
| A_55_P2010097 | Mup14 | major urinary protein 2, transcript variant 2, mRNA | NM_001045550 | -51.86 | 2.99E-09 | 0.311278 |
| A_55_P2061104 | Mup6 | major urinary protein 6, mRNA | NM_001081285 | -50.54 | 1.13E-08 | 0.182152 |
| A_66_P109802 | Ces3b | carboxylesterase 3B (Ces3b), transcript variant 2, mRNA | NM_001159415 | -46.93 | 1.68E-10 | 0.093895 |
| A_55_P2010093 | Mup4 | major urinary protein 4, mRNA | NM_008648 | -45.63 | 6.23E-11 | 0.802423 |
| A_55_P2051486 | Mup20 | major urinary protein 20, mRNA | NM_001012323 | -45.27 | 3.19E-11 | 0.743731 |
| A_55_P2025343 | Mup21 | major urinary protein 21, mRNA | NM_001009550 | -36.79 | 5.57E-08 | 0.070883 |
| A_55_P2061219 | Ces3a | carboxylesterase 3A, v1, mRNA | NM_198672 | -34.97 | 2.36E-11 | 0.561145 |
| A_55_P2103703 | Mup-ps21 | major urinary protein pseudogene 21, ncRNA | NG_031804 | -30.56 | 1.67E-09 | 0.188035 |
| A_55_P2113587 | Mup-ps12 | major urinary protein pseudogene 12, ncRNA | XR_140635 | -30.27 | 3.37E-10 | 0.418643 |
| A_55_P2144090 | Mup-ps3 | major urinary protein pseudogene 3, ncRNA | NG_065364 | -29.79 | 3.19E-11 | 0.630361 |
| A_55_P2092219 | Serpina9 | serine peptidase inhibitor, clade A (alpha-1 antiproteinase, antitrypsin), member 9, mRNA | NM_027997 | -28.74 | 6.51E-11 | **0.0124** |
| A_55_P1981197 | Mup-ps12 | major urinary protein pseudogene 12, ncRNA | XR_140635 | -26.90 | 3.31E-11 | 0.756958 |
| A_55_P2083233 | Mup-ps4 | major urinary protein pseudogene 4, ncRNA | NG_008456 | -23.61 | 3.25E-12 | 0.612633 |
| A_51_P269404 | Fmo3 | flavin containing monooxygenase 3, mRNA | NM_008030 | -21.59 | 2.54E-09 | 0.661619 |
| A_52_P73552 | A1bg | alpha-1-B glycoprotein, mRNA | NM_001081067 | -17.05 | 1.25E-07 | 0.702731 |
| A_55_P2046671 | Mup10 | major urinary protein 10, mRNA | NM_001122647.1 | -16.96 | 2.35E-06 | **0.00614** |
| A_55_P2059586 | Fmo3 | flavin containing monooxygenase 3, mRNA | NM_008030 | -15.58 | 3.83E-11 | 0.294809 |
| A_55_P1961466 | Dct | dopachrome tautomerase, mRNA | NM_010024 | -15.19 | 1.53E-05 | 0.105552 |
| A_51_P478881 | Ces4a | carboxylesterase 4A, mRNA | NM_146213 | -14.91 | 1.30E-08 | **0.01554** |
| A_55_P2033326 | Mup-ps7 | major urinary protein pseudogene 7, ncRNA | NG_008454 | -14.81 | 8.20E-12 | 0.560822 |
| A_55_P1963917 | Gm7298 | murinoglobulin 1 pseudogene, ncRNA | NAP093259-001 | -14.58 | 7.32E-11 | 0.612324 |
| A_52_P320193 | Clec2h | C-type lectin domain family 2, member h, mRNA | NM_053165 | -13.75 | 4.53E-07 | 0.095442 |
| A_52_P306357 | Prok1 | prokineticin 1, mRNA | NM_001357885.1 | -13.51 | 7.82E-08 | 0.184859 |
| A_55_P1989658 | Slco1a1 | solute carrier organic anion transporter family, member 1a1, mRNA | NM_013797 | -12.19 | 0.000511 | 0.072729 |
| A_55_P2063654 | Mup20 | major urinary protein 20, mRNA | NM_001012323 | -11.57 | 2.94E-11 | 0.894452 |
| A_55_P2003053 | Dct | dopachrome tautomerase, mRNA | NM_010024 | -10.58 | 3.55E-05 | 0.09929 |
| A_55_P1969861 | Zap70 | zeta-chain (TCR) associated protein kinase, mRNA | NM_009539 | -10.19 | 1.12E-07 | 0.545823 |
| A_66_P108979 | Olfr1535 | olfactory receptor 1535, mRNA | NM_207572 | -9.91 | 4.06E-06 | 0.076802 |
| A_55_P1963134 | Gm6135 | ncRNA | NR_166483.1 | -9.43 | 2.44E-08 | **0.00325** |
| A_51_P239737 | Pigr | polymeric immunoglobulin receptor, mRNA | NM_011082 | -9.13 | 4.07E-08 | 0.451726 |
| A_51_P257885 | Mmd2 | monocyte to macrophage differentiation-associated 2, mRNA | NM_175217 | -8.95 | 3.77E-05 | 0.065773 |
| A_55_P2162970 | Gm3734 | ncRNA | XR_877382.3 | -8.49 | 0.00012 | 0.937529 |
| A_55_P2129449 | Sult3a1 | sulfotransferase family 3A, member 1, mRNA | NM_020565 | -8.43 | 8.13E-09 | 0.386858 |
| A_55_P2153122 | Zap70 | zeta-chain (TCR) associated protein kinase, mRNA | NM_009539 | -8.25 | 3.97E-07 | 0.584371 |
| A_52_P402127 | Mup11 | major urinary protein 11, mRNA | NM_001126319 | -8.21 | 0.000101 | **0.01052** |
| A_55_P2141008 | Siglech | sialic acid binding Ig-like lectin H | XM_036152979.1 | -7.77 | 3.09E-06 | 0.05822 |
| A_51_P137452 | Cyp2g1 | cytochrome P450, family 2, subfamily g, polypeptide 1, mRNA | NM_013809 | -7.73 | 2.94E-06 | **0.02775** |
| A_55_P2303310 | C730036E19Rik | RIKEN cDNA C730036E19 gene (C730036E19Rik), non-coding RNA | NR_038011 | -7.58 | 4.28E-06 | 0.559114 |

**Supplementary Table ST6:** Top 50 genes **downregulated** in the liver of offspring nursed by α-casein deficient dams relative to offspring nursed by wildtype dams on **day 21**. 22 of the top 50 genes downregulated are major urinary protein (MUP) genes or pseudogenes (indicated in blue). These genes are clustered on mouse chromosome 4. They have no human homologue. 4 of the top 50 genes are serpin proteinase inhibitor genes or pseudogenes. 3 of the top 50 genes are carboxylesterases. The p-values of the gene expression changes on day 21 (p d21) and day 100 (p d100) are coloured by Microsoft Excel conditional formatting. The lowest p-values are in red, the highest p-values are in green. 7 of the top 50 genes downregulated at day 21 are still significantly changed at day 100 (shown in bold; 6 with p<0.05 and 1 with p<0.01). Genes expressing non-coding RNAs are marked in red in the GenBank column.

| **Agilent probe** | **GeneName** | **Description** | **GenBank** | **fold change d100** | **p d21** | **p d100** |
| --- | --- | --- | --- | --- | --- | --- |
| A_55_P2044653 | Cyp2b10 | cytochrome P450, family 2, subfamily b, polypeptide 10, v2, mRNA | NM_009999 | 9.82 | 0.889638 | 0.012762 |
| A_30_P01018914 | Gsta1 | Glutathione S-transferase A1, mRNA | NM_008181.3 | 7.91 | **0.00124** | 0.020703 |
| A_51_P367866 | Egr1 | early growth response 1, mRNA | NM_007913 | 6.25 | **0.04666** | 0.002523 |
| A_55_P2032946 | Gsta1 | Glutathione S-transferase A1, mRNA | NM_001243092 | 5.70 | **0.00049** | 0.024744 |
| A_51_P490023 | Tubb2a | tubulin, beta 2A class IIA, mRNA | NM_009450 | 5.56 | 0.055825 | 0.001043 |
| A_30_P01028766 | Gsta1 | Glutathione S-transferase A1, mRNA | NM_008181.3 | 5.54 | **0.00079** | 0.028504 |
| A_52_P235347 | Fgf21 | fibroblast growth factor 21, mRNA | NM_020013 | 5.21 | 0.302686 | 0.014366 |
| A_51_P238576 | Cyp4a14 | cytochrome P450, family 4, subfamily a, polypeptide 14, mRNA | NM_007822 | 4.50 | 0.140293 | 0.001689 |
| A_55_P2408588 | Arntl | aryl hydrocarbon receptor nuclear translocator-like, v1, mRNA | NM_007489 | 4.44 | **0.01738** | 0.000338 |
| A_55_P1968433 | Agpat9 | 1-acylglycerol-3-phosphate O-acyltransferase 9, v1, mRNA | NM_172715 | 4.34 | **0.04988** | 0.001275 |
| A_55_P1960735 | Gdf15 | growth differentiation factor 15, mRNA | NM_011819 | 4.33 | 0.41789 | 0.000923 |
| A_51_P269792 | Rad51b | RAD51 homolog B, v1, mRNA | NM_009014 | 4.29 | 0.187788 | 8.94E-05 |
| A_55_P1991841 | Slc22a27 | solute carrier family 22, member 27, mRNA | NM_134256 | 4.24 | 0.735891 | 0.001923 |
| A_51_P142923 | Chka | choline kinase alpha, transcript variant 1, mRNA | NM_013490 | 4.05 | 0.074682 | 0.000645 |
| A_52_P381484 | Spon2 | spondin 2, extracellular matrix protein, mRNA | NM_133903 | 3.85 | 0.24152 | 2.96E-05 |
| A_55_P2165091 | Acnat2 | acyl-coenzyme A amino acid N-acyltransferase 2, mRNA | NM_145368 | 3.67 | 0.213501 | 3.36E-05 |
| A_55_P2075263 | Acnat2 | acyl-coenzyme A amino acid N-acyltransferase 2, mRNA | NM_145368 | 3.64 | 0.492583 | 6.21E-06 |
| A_55_P2102065 | Gsta5 | Glutathione S-Transferase Alpha 5, mRNA | NM_001122660.1 | 3.51 | **0.00016** | 0.033683 |
| A_55_P2077783 | Tubb2a-ps2 | tubulin, beta 2a, pseudogene 2, non-coding RNA | NR_003964 | 3.35 | 0.078409 | 0.004176 |
| A_51_P363947 | Cdkn1a | cyclin-dependent kinase inhibitor 1A (P21), v1, mRNA | NM_007669 | 3.31 | 0.305235 | 0.027739 |
| A_52_P140005 | Nipal1 | NIPA-like domain containing 1, mRNA | NM_001081205 | 3.29 | **0.00314** | 0.000473 |
| A_51_P144531 | Slc22a29 | solute carrier family 22. member 29, mRNA | NM_172776 | 3.22 | 0.429134 | 0.002624 |
| A_52_P338066 | Ubd | ubiquitin D, mRNA | NM_023137 | 3.22 | 0.075045 | 0.047589 |
| A_51_P267354 | Lrfn3 | leucine rich repeat and fibronectin type III domain containing 3, mRNA | NM_175478 | 3.18 | **0.02339** | 0.000106 |
| A_55_P2115955 | Raet1e | retinoic acid early transcript 1E, mRNA | NM_198193 | 3.07 | **0.03592** | 0.012133 |
| A_52_P329367 | Chka | choline kinase alpha, mRNA | NM_001410133.1 | 3.05 | 0.092867 | 0.003313 |
| A_51_P467448 | Pif1 | PIF1 5'-to-3' DNA helicase homolog, mRNA | NM_172453 | 3.00 | 0.490383 | 2.27E-05 |
| A_55_P2030752 | Nedd4l | neural precursor cell expressed, developmentally down-regulated gene 4-like, v2, mRNA | NM_031881 | 2.97 | 0.308284 | 2.97E-05 |
| A_55_P2170454 | Gsta2 | glutathione S-transferase, alpha 2 (Yc2), mRNA | NM_008182 | 2.92 | **3.88E-5** | 0.022482 |
| A_55_P1956223 | Chka | choline kinase alpha, v3, mRNA | NM_001271496 | 2.90 | 0.200612 | 0.002555 |
| A_51_P286737 | Ccl2 | chemokine (C-C motif) ligand 2, mRNA | NM_011333 | 2.88 | 0.3482 | 0.048404 |
| A_55_P2007601 | Sftpd | surfactant associated protein D, mRNA | NM_009160 | 2.82 | 0.760227 | 0.018272 |
| A_55_P2024046 | Slc16a5 | solute carrier family 16 (monocarboxylic acid transporters), member 5, mRNA | NM_001080934 | 2.82 | **0.04731** | 0.000816 |
| A_55_P2038358 | Acot1 | acyl-CoA thioesterase 1, mRNA | NM_012006 | 2.80 | **0.04241** | 0.00763 |
| A_51_P355301 | Cyp3a11 | cytochrome P450, family 3, subfamily a, polypeptide 11, mRNA | NM_007818 | 2.80 | 0.167079 | 0.001721 |
| A_52_P382149 | Cyp26a1 | cytochrome P450, family 26, subfamily a, polypeptide 1, mRNA | NM_007811 | 2.79 | 0.774035 | 0.006957 |
| A_51_P228295 | Mpzl1 | myelin protein zero-like 1, v2, mRNA | NM_001001880 | 2.78 | **0.00059** | 4.69E-05 |
| A_51_P155196 | Abtb2 | ankyrin repeat and BTB (POZ) domain containing 2, mRNA | NM_178890 | 2.76 | **0.00043** | 7.56E-05 |
| A_55_P2080956 | Chka | choline kinase alpha, v1, mRNA | NM_013490 | 2.74 | 0.140702 | 0.005011 |
| A_55_P2387665 | Slc10a2 | solute carrier family 10, member 2, mRNA | NM_011388.3 | 2.73 | **0.01102** | 0.002939 |
| A_51_P495269 | Lor | loricrin, mRNA | NM_008508 | 2.64 | 0.984492 | 0.000416 |
| A_55_P2110497 | Ddc | dopa decarboxylase, v1, mRNA | NM_001190448 | 2.64 | 0.246705 | 0.0002 |
| A_51_P287198 | Krt23 | keratin 23, mRNA | NM_033373 | 2.63 | 0.680179 | 0.016094 |
| A_66_P106760 | Adam32 | a disintegrin and metallopeptidase domain 32, mRNA | NM_153397 | 2.62 | 0.315527 | 0.011144 |
| A_55_P2159555 | Adamts4 | ADAM metallopeptidase with thrombospondin type 1 motif 4, mRNA | NM_172845.3 | 2.62 | 0.500747 | 0.007407 |
| A_52_P413947 | Mthfr | 5,10-methylenetetrahydrofolate reductase, v2, mRNA | NM_010840 | 2.62 | 0.468912 | 0.000183 |
| A_66_P114381 | Ypel2 | yippee-like 2 (Drosophila), mRNA | NM_001005341 | 2.62 | 0.710124 | 0.00046 |
| A_51_P304683 | Clpx | caseinolytic peptidase X (E.coli), transcript variant 1, mRNA | NM_011802 | 2.61 | 0.142074 | 9.09E-06 |
| A_55_P2043627 | Fam89a | family with sequence similarity 89, member A, mRNA | NM_001081120 | 2.61 | 0.60851 | 1.22E-05 |

**Supplementary Table ST7:** Top 50 genes **upregulated** in the liver of offspring nursed by α-casein deficient dams relative to offspring nursed by wildtype dams at **day 100**. Five of the top 50 genes upregulated are glutathione S-transferase genes (highlighted in blue). Four of the top 50 genes upregulated encode Cytochrome P450 enzymes (highlighted in yellow). All gene expression changes between WT nursed and KO nursed animals are significant on day 100. The p-values of the gene expression changes on day 21 (p d21) and day 100 (p d100) are coloured by Microsoft Excel conditional formatting. The lowest p-values are in red, the highest p-values are in green. 17 of the 50 genes are also changed significantly on day 21 (13 with p<0.05 and 8 with p<0.001). Genes expressing non-coding RNAs are marked in red in the GenBank column.

| **Agilent ID** | **GeneName** | **Description** | **Genbank** | **fold change d100** | **p.21** | **p.100** |
| --- | --- | --- | --- | --- | --- | --- |
| A_55_P2111980 | Hsd3b5 | hydroxy-delta-5-steroid dehydrogenase, mRNA | NM_008295 | -6.61 | **8.25E-5** | 0.026286 |
| A_55_P2075127 | Pax2 | paired box gene 2, mRNA | NM_011037 | -6.38 | 0.062246 | 0.028563 |
| A_55_P1985788 | Usp2 | ubiquitin specific peptidase 2, v3, mRNA | NM_198092 | -6.09 | 0.625746 | 0.000238 |
| A_51_P491667 | Derl3 | Der1-like domain family, member 3, mRNA | NM_024440 | -4.95 | **0.00475** | 0.000404 |
| A_30_P01024647 | Hnrnpr | heterogeneous nuclear ribonucleoprotein R, tvX2, ncRNA. | XR_004941997.1 | -4.74 | 0.831257 | 0.00213 |
| A_52_P63343 | Ciart | circadian associated repressor of transcription, v1, mRNA | NM_001033302 | -4.60 | **0.02632** | 0.000536 |
| A_51_P493987 | Moxd1 | Mus musculus monooxygenase, DBH-like 1 (Moxd1), mRNA [NM_021509] | NM_021509 | -4.59 | 0.862309 | 1.28E-05 |
| A_30_P01017959 | Gm30233 | predicted gene, 30233, vX1, ncRNA | XR_004936379.1 | -3.93 | 0.946639 | 0.002862 |
| A_55_P2051159 | Upp2 | uridine phosphorylase 2, mRNA | NM_029692 | -3.89 | **0.03023** | 0.000135 |
| A_55_P2032079 | Dbp | D site albumin promoter binding protein, mRNA | NM_016974 | -3.65 | **0.01661** | 0.005001 |
| A_55_P2018666 | Thrsp | thyroid hormone responsive, mRNA | NM_009381 | -3.64 | **0.02722** | 0.021702 |
| A_52_P259817 | Upp2 | uridine phosphorylase 2, mRNA | NM_029692 | -3.63 | 0.181888 | 0.000194 |
| A_51_P152990 | Grem2 | gremlin 2 homolog, cysteine knot superfamily, mRNA | NM_011825 | -3.60 | 0.544206 | 0.000294 |
| A_55_P2164075 | Gm10804 | predicted gene 10804, v2, non-coding RNA | NR_040533 | -3.59 | 0.703426 | 0.012156 |
| A_55_P2046671 | Mup10 | major urinary protein 10, mRNA | NM_001122647 | -3.49 | **2.35E-6** | 0.006144 |
| A_51_P461429 | Cyp7b1 | cytochrome P450, family 7, subfamily b, polypeptide 1, mRNA | NM_007825 | -3.47 | 0.682947 | 0.000518 |
| A_52_P566840 | Gpr110 | G protein-coupled receptor 110, mRNA | NM_133776 | -3.47 | **0.01221** | 0.002962 |
| A_55_P2167269 | Pcsk4 | proprotein convertase subtilisin/kexin type 4, mRNA | NM_008793 | -3.45 | 0.481289 | 0.000439 |
| A_55_P2032081 | Dbp | D site albumin promoter binding protein, mRNA | NM_016974 | -3.38 | **0.01062** | 0.006646 |
| A_52_P84027 | Cyp7a1 | cytochrome P450, family 7, subfamily a, polypeptide 1, mRNA | NM_007824 | -3.36 | 0.244144 | 0.000197 |
| A_55_P1978770 | E030018B13Rik | RIKEN cDNA E030018B13 gene (Gm53391), ncRNA | NM_001256311 | -3.32 | 0.725925 | 9.91E-07 |
| A_55_P1976351 | Gpcpd1 | glycerophosphocholine phosphodiesterase GDE1 homolog, v4, mRNA | NM_001042672 | -3.32 | 0.63548 | 2.06E-05 |
| A_52_P402127 | Mup9 | major urinary protein 9, mRNA | NM_001126319 | -3.28 | **0.0001** | 0.010519 |
| A_55_P2212161 | 2310034O05Rik | predicted gene, long non-coding RNA | AK009622 | -3.28 | 0.092048 | 0.000635 |
| A_66_P130647 | Onecut1 | one cut domain, family member 1, mRNA | NM_008262 | -3.22 | **0.0015** | 0.012821 |
| A_66_P120125 | Nrep | neuronal regeneration related protein, v1, mRNA | NM_053078 | -3.12 | **0.00097** | 0.000197 |
| A_55_P2399499 | B930025P03Rik | RIKEN cDNA B930025P03 gene, non-coding RNA | NR_040705 | -3.12 | 0.972193 | 0.009364 |
| A_55_P1966029 | A930033H14Rik | RIKEN cDNA A930033H14 gene, misc_RNA | XR_105403 | -2.94 | 0.178083 | 0.000109 |
| A_51_P281778 | Igsf23 | immunoglobulin superfamily, member 23, mRNA | NM_027308 | -2.93 | **3.73E-6** | 2.60E-05 |
| A_55_P2092501 | Med1 | mediator complex subunit 1, v2, mRNA | NM_134027 | -2.84 | 0.292378 | 0.0011 |
| A_51_P453043 | Aacs | acetoacetyl-CoA synthetase, mRNA | NM_030210 | -2.83 | **0.02725** | 0.006652 |
| A_51_P514405 | Slc2a5 | solute carrier family 2 (facilitated glucose transporter), member 5, mRNA | NM_019741 | -2.81 | 0.352659 | 0.007623 |
| A_66_P134394 | Pde6c | phosphodiesterase 6C, cGMP specific, cone, alpha prime, v1, mRNA | NM_033614 | -2.75 | **0.00986** | 0.015048 |
| A_66_P136186 | Wee1 | WEE 1 homolog 1, mRNA | NM_009516 | -2.71 | **0.03092** | 0.000651 |
| A_55_P2255449 | Map2k6 | mitogen-activated protein kinase kinase 6, mRNA | AK033778 | -2.69 | 0.306479 | 0.001131 |
| A_51_P256093 | Map2k6 | mitogen-activated protein kinase kinase 6, mRNA | NM_011943 | -2.66 | 0.488019 | 0.001501 |
| A_55_P1981195 | Mup-ps12 | major urinary protein, pseudogene 12, miscRNA | XR_140635 | -2.66 | 0.084279 | 0.015677 |
| A_55_P2387915 | Gm10804 | predicted gene 10804, v1, non-coding RNA | NR_040532 | -2.64 | 0.961115 | 0.021872 |
| A_55_P1973447 | Ybx2 | Y box protein 2, mRNA | NM_016875 | -2.63 | 0.328923 | 0.002843 |
| A_55_P2041668 | Foxl2 | forkhead box L2, mRNA | NM_012020 | -2.63 | 0.907937 | 0.002968 |
| A_55_P2016014 | Cpsf4l | cleavage and polyadenylation specific factor 4-like, v3, mRNA | NM_001164532 | -2.62 | 0.808105 | 0.028287 |
| A_55_P2089488 | Coq10b | coenzyme Q10 homolog B, nuclear gene encoding mitochondrial protein, v1, mRNA | NM_001039710 | -2.62 | 0.068452 | 1.33E-05 |
| A_55_P1994733 | Tmem14a | transmembrane protein 14A | NM_001290679 | -2.61 | 0.413755 | 0.000875 |
| A_55_P1954393 | Susd4 | sushi domain containing 4, mRNA | NM_144796 | -2.60 | 0.078619 | 0.011958 |
| A_52_P366525 | Coq10b | coenzyme Q10 homolog B, nuclear gene encoding mitochondrial protein, v1, mRNA | NM_001039710 | -2.60 | 0.057094 | 5.03E-06 |
| A_55_P2185900 | Nrg4 | neuregulin 4, mRNA | NM_032002 | -2.57 | 0.371356 | 4.87E-05 |
| A_52_P329398 | Atp12a | ATPase, H+/K+ transporting, nongastric, alpha polypeptide, mRNA | NM_138652 | -2.56 | 0.623697 | 0.002663 |
| A_55_P2082658 | Gm31105 | predicted gene, long non-coding RNA | ENSMUSG00000109587 | -2.53 | 0.689748 | 0.000399 |
| A_55_P2159264 | Lifr | leukemia inhibitory factor receptor, v2, mRNA | NM_001113386 | -2.51 | **5.00E-7** | 0.000393 |

**Supplementary Table ST8:** Top 50 genes **downregulated** in the liver of offspring nursed by α-casein deficient dams relative to offspring nursed by wildtype dams on **day 100**. 3 of the top 50 genes downregulated are major urinary protein MUP genes or pseudogenes (indicated in blue). The p-values of the gene expression changes on day 21 (p d21) and day 100 (p d100) are coloured by Microsoft Excel conditional formatting. 7 of the top 50 genes downregulated at day 21 are still significantly changed at day 100 (shown in bold; 6 with p<0.05 and 1 with p<0.01). Genes expressing non-coding RNAs are marked in red in the GenBank column.

| **Term** | **Library** | **p-value** |
| --- | --- | --- |
| Palbociclib Down | LINCS_L1000_Chem_Pert_Consensus_Sigs | 9.50E-52 |
| C646 Down | LINCS_L1000_Chem_Pert_Consensus_Sigs | 2.60E-39 |
| SAR405838 Down | LINCS_L1000_Chem_Pert_Consensus_Sigs | 1.90E-36 |
| AMG-232 Down | LINCS_L1000_Chem_Pert_Consensus_Sigs | 3.51E-35 |
| R-547 Down | LINCS_L1000_Chem_Pert_Consensus_Sigs | 2.81E-33 |
| RPL19 Down | LINCS_L1000_CRISPR_KO_Consensus_Sigs | 1.31E-28 |
| HSPA5 Down | LINCS_L1000_CRISPR_KO_Consensus_Sigs | 2.65E-27 |
| CDK4 Down | LINCS_L1000_CRISPR_KO_Consensus_Sigs | 1.94E-26 |
| IMP4 Down | LINCS_L1000_CRISPR_KO_Consensus_Sigs | 2.28E-26 |
| XPO1 Down | LINCS_L1000_CRISPR_KO_Consensus_Sigs | 1.75E-23 |
| microtubule cytoskeleton organization involved in mitosis (GO:1902850) | GO_Biological_Process_2021 | 1.16E-16 |
| mitotic spindle organization (GO:0007052) | GO_Biological_Process_2021 | 1.16E-13 |
| DNA metabolic process (GO:0006259) | GO_Biological_Process_2021 | 5.28E-11 |
| Cell cycle | KEGG_2021_Human **[SG]** | 9.53E-10 |
| DNA replication (GO:0006260) | GO_Biological_Process_2021 | 1.13E-09 |
| DNA strand elongation involved in DNA replication (GO:0006271) | GO_Biological_Process_2021 | 1.67E-08 |
| abnormal mitosis MP:0004046 | MGI_Mammalian_Phenotype_Level_4_2021 | 2.32E-08 |
| chromosomal instability MP:0008866 | MGI_Mammalian_Phenotype_Level_4_2021 | 6.48E-08 |
| Glutathione metabolism | KEGG_2021_Human **[SG]** | 7.12E-08 |
| Metabolism of xenobiotics by cytochrome P450 | KEGG_2021_Human **[SG]** | 1.24E-07 |
| DNA replication | KEGG_2021_Human **[SG]** | 2.16E-07 |
| Fluid shear stress and atherosclerosis | KEGG_2021_Human **[SG]** | 0.000002 |
| increased lymphoma incidence MP:0012431 | MGI_Mammalian_Phenotype_Level_4_2021 | 9.914E-06 |
| decreased tumor latency MP:0010308 | MGI_Mammalian_Phenotype_Level_4_2021 | 0.00001 |
| abnormal cell nucleus morphology MP:0003111 | MGI_Mammalian_Phenotype_Level_4_2021 | 0.00001 |

**Supplementary table ST9:** Enrichment analysis of the list of genes **upregulated** in the liver of offspring nursed by α-casein deficient dams at **day 21**. The gene list was analysed using the EnricherKG, ShinyGo and Metascape pathway analysis programs. The top 20 enriched pathways (using the KEGG 2021 pathways, LINCS L1000 CRISPR, LINCS L1000 Chemical Perturbation, GO Biological Process 2021 and MGI Mammalian Phenotype 2021 databases) identified in EnrichrKG are shown. Pathways also identified by ShinyGo are indicated by **[SG]** in the library column. The p-values of enrichment are shown.

| **Term** | **Library** | **p-value** |
| --- | --- | --- |
| APO-866 Down | LINCS_L1000_Chem_Pert_Consensus_Sigs | 4.66E-09 |
| Terreic-Acid Down | LINCS_L1000_Chem_Pert_Consensus_Sigs | 1.11E-06 |
| SGI-1776 Up | LINCS_L1000_Chem_Pert_Consensus_Sigs | 1.11E-06 |
| ketone body biosynthetic process (GO:0046951) | GO_Biological_Process_2021 | 2.09E-06 |
| ketone body metabolic process (GO:1902224) | GO_Biological_Process_2021 | 5.37E-06 |
| RIPOR2 Down | LINCS_L1000_CRISPR_KO_Consensus_Sigs | 6.1E-06 |
| TGX-115 Down | LINCS_L1000_Chem_Pert_Consensus_Sigs | 6.1E-06 |
| BIBU-1361 Up | LINCS_L1000_Chem_Pert_Consensus_Sigs | 6.37E-06 |
| ACTR3 Down | LINCS_L1000_CRISPR_KO_Consensus_Sigs | 2.96E-05 |
| ROS1 Up | LINCS_L1000_CRISPR_KO_Consensus_Sigs | 3.2E-05 |
| SLC2A3 Down | LINCS_L1000_CRISPR_KO_Consensus_Sigs | 3.33E-05 |
| ODC1 Up | LINCS_L1000_CRISPR_KO_Consensus_Sigs | 0.000107 |
| peptidyl-tyrosine phosphorylation (GO:0018108) | GO_Biological_Process_2021 | 0.000145 |
| Synthesis and degradation of ketone bodies | KEGG_2021_Human | 0.000172 |
| enzyme linked receptor protein signaling pathway (GO:0007167) | GO_Biological_Process_2021 | 0.000227 |
| Butanoate metabolism | KEGG_2021_Human **[SG, MS]** | 0.000286 |
| short hair MP:0000417 | MGI_Mammalian_Phenotype_Level_4_2021 | 0.00031 |
| peptidyl-tyrosine modification (GO:0018212) | GO_Biological_Process_2021 | 0.000466 |
| abnormal long bone epiphyseal plate proliferative zone MP:0003662 | MGI_Mammalian_Phenotype_Level_4_2021 | 0.000548 |
| sensorineural hearing loss MP:0004740 | MGI_Mammalian_Phenotype_Level_4_2021 | 0.000685 |
| abnormal breathing pattern MP:0001951 | MGI_Mammalian_Phenotype_Level_4_2021 | 0.000849 |
| decreased activity of thyroid gland MP:0003503 | MGI_Mammalian_Phenotype_Level_4_2021 | 0.000918 |
| Steroid biosynthesis | KEGG_2021_Human **[SG, MS]** | 0.001499 |
| Ovarian steroidogenesis | KEGG_2021_Human **[SG, MS]** | 0.002833 |
| Complement and coagulation cascades | KEGG_2021_Human **[SG, MS]** | 0.003051 |

**Supplementary table ST10:** Enrichment analysis of the list of genes **downregulated** in the liver of offspring nursed by α-casein deficient dams at **day 21**. The gene list was analysed using the EnricherKG, ShinyGo and Metascape pathway analysis programs. The top 20 enriched pathways (using the KEGG 2021 pathways, LINCS L1000 CRISPR, LINCS L1000 Chemical Perturbation, GO Biological Process 2021 and MGI Mammalian Phenotype 2021 databases) identified in EnrichrKG. Pathways also identified by ShinyGo **[SG]** and/or Metascape **[MS]** are indicated in the library column. The p-values of enrichment are shown.

| **Term** | **Library** | **p-value** |
| --- | --- | --- |
| Semagacestat Down | LINCS_L1000_Chem_Pert_Consensus_Sigs | 1.53E-08 |
| SKF-81297 Down | LINCS_L1000_Chem_Pert_Consensus_Sigs | 1.60E-08 |
| Ingenol Up | LINCS_L1000_Chem_Pert_Consensus_Sigs | 1.83E-08 |
| PLS3 Down | LINCS_L1000_CRISPR_KO_Consensus_Sigs | 1.83E-08 |
| NPC26 Up | LINCS_L1000_Chem_Pert_Consensus_Sigs | 1.23E-07 |
| AG-1024 Up | LINCS_L1000_Chem_Pert_Consensus_Sigs | 1.28E-07 |
| HPN Down | LINCS_L1000_CRISPR_KO_Consensus_Sigs | 1.39E-07 |
| DAZL Down | LINCS_L1000_CRISPR_KO_Consensus_Sigs | 1.45E-07 |
| PROM1 Down | LINCS_L1000_CRISPR_KO_Consensus_Sigs | 1.52E-07 |
| SUZ12 Up | LINCS_L1000_CRISPR_KO_Consensus_Sigs | 1.58E-07 |
| increased susceptibility to induced thrombosis MP:0031121 | MGI_Mammalian_Phenotype_Level_4_2021 | 6.77E-06 |
| abnormal vascular wound healing MP:0004883 | MGI_Mammalian_Phenotype_Level_4_2021 | 1.27E-05 |
| increased liver triglyceride level MP:0009355 | MGI_Mammalian_Phenotype_Level_4_2021 | 7.37E-05 |
| increased neuron number MP:0008947 | MGI_Mammalian_Phenotype_Level_4_2021 | 7.81E-05 |
| increased bone marrow cell number MP:0000321 | MGI_Mammalian_Phenotype_Level_4_2021 | 8.62E-05 |
| positive regulation of low-density lipoprotein receptor activity (GO:1905599) | GO_Biological_Process_2021 | 0.000319 |
| Fatty acid elongation | KEGG_2021_Human **[SG, MS]** | 0.000477 |
| Biosynthesis of unsaturated fatty acids | KEGG_2021_Human **[SG, MS]** | 0.000477 |
| negative regulation of neuron apoptotic process (GO:0043524) | GO_Biological_Process_2021 **[MS]** | 0.000725 |
| positive regulation of neurogenesis (GO:0050769) | GO_Biological_Process_2021 | 0.000764 |
| very long-chain fatty acid metabolic process (GO:0000038) | GO_Biological_Process_2021 **[MS]** | 0.000869 |
| stress-induced premature senescence (GO:0090400) | GO_Biological_Process_2021 | 0.000882 |
| PI3K-Akt signaling pathway | KEGG_2021_Human **[SG, MS]** | 0.00419 |
| MAPK signaling pathway | KEGG_2021_Human **[MS]** | 0.006805 |
| Homologous recombination | KEGG_2021_Human **[SG]** | 0.02285 |

**Supplementary table ST11:** Enrichment analysis of the list of genes **upregulated** in the liver of offspring nursed by α-casein deficient dams at **day 100**. The gene list was analysed using the EnricherKG, ShinyGo and Metascape pathway analysis programs. The top 20 enriched pathways (using the KEGG 2021 pathways, LINCS L1000 CRISPR, LINCS L1000 Chemical Perturbation, GO Biological Process 2021 and MGI Mammalian Phenotype 2021 databases were used to identify enriched pathways in EnrichrKG. Pathways also identified by ShinyGo **[SG]** and/or Metascape **[MS]** are indicated in the library column. The p-values of enrichment are shown.

| **Term** | **Library** | **p-value** |
| --- | --- | --- |
| shortened circadian behavior period MP:0020470 | MGI_Mammalian_Phenotype_Level_4_2021 | 3.8E-06 |
| enhanced conditioned place preference behavior MP:0009713 | MGI_Mammalian_Phenotype_Level_4_2021 | 4.85E-06 |
| entrainment of circadian clock by photoperiod (GO:0043153) | GO_Biological_Process_2021 **[MS]** | 6.22E-06 |
| photoperiodism (GO:0009648) | GO_Biological_Process_2021 **[MS]** | 7.23E-06 |
| enhanced behavioral response to cocaine MP:0009754 | MGI_Mammalian_Phenotype_Level_4_2021 | 5.72E-05 |
| cellular response to hexose stimulus (GO:0071331) | GO_Biological_Process_2021 **[MS]** | 0.000189 |
| abnormal basal metabolism MP:0008961 | MGI_Mammalian_Phenotype_Level_4_2021 | 0.000297 |
| Circadian rhythm | KEGG_2021_Human **[SG]** | 0.000362 |
| abnormal circadian sleep/wake cycle MP:0020478 | MGI_Mammalian_Phenotype_Level_4_2021 | 0.000414 |
| androgen biosynthetic process (GO:0006702) | GO_Biological_Process_2021 | 0.000706 |
| positive regulation of cell activation (GO:0050867) | GO_Biological_Process_2021 | 0.00088 |
| GABRA3 Up | LINCS_L1000_CRISPR_KO_Consensus_Sigs | 0.000894 |
| Primary bile acid biosynthesis | KEGG_2021_Human **[SG]** | 0.002606 |
| Steroid hormone biosynthesis | KEGG_2021_Human **[MS, SG]** | 0.002626 |
| Promethazine Down | LINCS_L1000_Chem_Pert_Consensus_Sigs | 0.004586 |
| ENTPD1 Down | LINCS_L1000_CRISPR_KO_Consensus_Sigs | 0.004916 |
| CORO1A Up | LINCS_L1000_CRISPR_KO_Consensus_Sigs | 0.004916 |
| DPF1 Down | LINCS_L1000_CRISPR_KO_Consensus_Sigs | 0.004916 |
| MGAM Up | LINCS_L1000_CRISPR_KO_Consensus_Sigs | 0.005001 |
| Olprinone Up | LINCS_L1000_Chem_Pert_Consensus_Sigs | 0.005087 |
| Enobosarm Down | LINCS_L1000_Chem_Pert_Consensus_Sigs | 0.005087 |
| BW-373U86 Down | LINCS_L1000_Chem_Pert_Consensus_Sigs | 0.005174 |
| Medrysone Up | LINCS_L1000_Chem_Pert_Consensus_Sigs | 0.005174 |
| Circadian entrainment | KEGG_2021_Human **[MS]** | 0.009581 |
| Hepatitis B | KEGG_2021_Human | 0.03674 |

**Supplementary table ST12:** Enrichment analysis of the list of genes **downregulated** in the liver of offspring nursed by α-casein deficient dams at **day 100**. The gene list was analysed using the EnricherKG, ShinyGo and Metascape pathway analysis programs. The top 20 enriched pathways (using the KEGG 2021 pathways, LINCS L1000 CRISPR, LINCS L1000 Chemical Perturbation, GO Biological Process 2021 and MGI Mammalian Phenotype 2021 databases were used to identify enriched pathways in EnrichrKG. Pathways also identified by ShinyGo **[SG]** and/or Metascape **[MS]** are indicated in the library column. The p-values of enrichment are shown.
